# The small GTPase MglA together with the TPR domain protein SgmX stimulates type IV pili formation in *M. xanthus*

**DOI:** 10.1073/pnas.2004722117

**Published:** 2020-09-08

**Authors:** Anna Potapova, Luís Antonío Menezes Carreira, Lotte Søgaard-Andersen

**Affiliations:** ^a^Department of Ecophysiology, Max Planck Institute for Terrestrial Microbiology, 35043 Marburg, Germany

**Keywords:** type IV pili, bacterial motility, PilB ATPase, bacterial polarity, MglA GTPase

## Abstract

Bacteria can move across surfaces using type IV pili (T4P), which undergo cycles of extension, adhesion, and retraction. The T4P localization pattern varies between species; however, the underlying mechanisms are largely unknown. In the rod-shaped *Myxococcus xanthus* cells, T4P localize at the leading cell pole. As cells reverse their direction of movement, T4P are disassembled at the old leading pole and then form at the new leading pole. Thus, cells can form T4P at both poles but engage only one pole at a time in T4P formation. Here, we address how this T4P unipolarity is realized. We demonstrate that the small Ras-like GTPase MglA stimulates T4P formation in its GTP-bound state by direct interaction with the tetratricopeptide repeat (TPR) domain-containing protein SgmX. SgmX, in turn, is important for polar localization of the T4P extension ATPase PilB. The cognate MglA GTPase activating protein (GAP) MglB, which localizes mainly to the lagging cell pole, indirectly blocks T4P formation at this pole by stimulating the conversion of MglA-GTP to MglA-GDP. Based on these findings, we propose a model whereby T4P unipolarity is accomplished by stimulation of T4P formation at the leading pole by MglA-GTP and SgmX and indirect inhibition of T4P formation at the lagging pole by MglB due to its MglA GAP activity. During reversals, MglA, SgmX, and MglB switch polarity, thus laying the foundation for T4P formation at the new leading pole and inhibition of T4P formation at the new lagging pole.

## Significance

Many bacteria move across surfaces using type IV pili (T4P). The piliation pattern varies between species; however, the underlying mechanisms governing these patterns remain largely unknown. Here, we demonstrate that in the rod-shaped *Myxococcus xanthus* cells, the unipolar formation of T4P at the leading cell pole is the result of stimulation by the small GTPase MglA together with the effector protein SgmX, while MglB, the cognate MglA GTPase activating protein (GAP) that localizes to the lagging cell pole, blocks this stimulation at the lagging pole due to its GAP activity. During reversals, MglA/SgmX and MglB switch polarity, laying the foundation for T4P formation at the new leading cell pole and inhibition of T4P formation at the former leading cell pole.

In bacteria, motility is important for fitness, colonization of diverse habitats, virulence, and biofilm formation (1). One type of bacterial motility depends on type IV pili (T4P). T4P are cell surface filaments that also function in surface adhesion, surface sensing, host cell interaction, biofilm formation, virulence, and DNA uptake (2–5). T4P are highly dynamic and undergo cycles of extension, surface adhesion, and retraction (6, 7), with retractions generating a force exceeding 150 pN per T4P and pulling cells across surfaces (8, 9).

In Gram-negative bacteria, the type IV pilus machine (T4PM) that drives the extension/retraction cycles consists of 10 highly conserved proteins, referred to as the core proteins, that form a complex that spans the cell envelope (3, 4, 10–12) (*SI Appendix*, Fig. S1*A*). The hexameric ATPases PilB and PilT (13–16) power pilus extension and retraction, respectively (17, 18) and associate with the base of the T4PM in a mutually exclusive fashion (10). All core proteins are important for T4P extension except for PilT, which is only important for retraction. In addition, accessory proteins, which are much less conserved than the core proteins, regulate T4PM function and localization. FimX and PilZ in the gamma-proteobacteria *Pseudomonas aeruginosa* and *Xanthomonas axonopodis* pv. citri are the best understood among these proteins. In *P. aeruginosa*, FimX binds bis-(3′-5′)-cyclic dimeric guanosine monophosphate (c-di-GMP) and interacts directly with PilB to stimulate T4P formation and localization at the leading cell pole (19–22), while it remains unknown how PilZ stimulates T4P formation (23). In *X. axonopodis* pv. citri, FimX binds c-di-GMP and interacts with PilZ, which in turn, interacts with PilB to stimulate T4P formation (24). In *P. aeruginosa*, FimW also binds c-di-GMP and stimulates T4P formation by an unknown mechanism (25). By contrast, T4PM function and T4P localization in the delta-proteobacteria *Bdellovibrio bacteriovorus* and *Myxococcus xanthus* as well as in *Thermus thermophilus* involve the small Ras-like GTPase MglA. In *B. bacteriovorus*, MglA is important for T4P formation by an unknown mechanism (26); in *T. thermophilus*, MglA is not important for T4P formation but by an unknown mechanism for T4P localization to only one of the cell poles (27), and in *M. xanthus*, MglA is essential for T4P-dependent motility (28–31). Here, we address the function of MglA in T4PM function and localization in *M. xanthus*.

The rod-shaped *M. xanthus* cells move across surfaces in the direction of their long axis with defined leading and lagging cell poles using two motility systems, one for gliding and one for T4P-dependent motility (32). T4P are only present at the leading pole (33). T4PMs consisting of seven of the core proteins (PilQ, TsaP, PilP, PilO, PilN, PilM, and PilC) (*SI Appendix*, Fig. S1*A*) are present at both cell poles (10, 34–37). By contrast, PilB and PilT predominantly localize to the leading and lagging pole, respectively, and PilT only occasionally accumulates at the leading pole, stimulating retractions (18, 35). MglA is essential for both motility systems and cycles between the active MglA-GTP state, which stimulates motility by interacting with downstream effectors (38, 39), and the inactive MglA-GDP state (30, 31, 40). The MglA GTPase cycle is regulated by the RomR/RomX complex, which has guanine nucleotide exchange factor (GEF) activity and stimulates formation of the active GTP-bound form (41), and the GTPase activating protein (GAP) MglB, which stimulates the low intrinsic GTPase activity of MglA to convert the GTP bound to the inactive GDP-bound state (30, 31, 40). All four proteins localize to the poles (30, 31, 41–44). MglA-GTP primarily localizes to the leading pole, while MglA-GDP is diffusely distributed. MglB, RomR, and RomX localize in bipolar, asymmetric patterns with the large cluster at the lagging pole. The RomR/RomX GEF complex recruits MglA-GTP to the leading pole by stimulating formation of MglA-GTP and by directly binding MglA-GTP. MglB excludes MglA-GTP from the lagging pole by converting MglA-GTP to MglA-GDP. Among the four proteins, only MglA is essential for T4P-dependent motility (30, 31, 41, 45).

*M. xanthus* cells rapidly reverse their direction of movement in response to signaling by the Frz chemosensory system (46). Reversals entail a switch in T4P polarity, with T4P only forming at the new leading pole after a reversal (47). Thus, T4P can form at both poles, but only one pole at a time engages in T4P formation. How T4P unipolarity is established is not known. During the Frz-induced reversals, MglA, MglB, RomR, and RomX (30, 31, 41, 42) as well as PilB and PilT switch poles (34–36).

Here, we demonstrate that MglA-GTP stimulates T4P formation via direct interaction with the tetratricopeptide repeat (TPR) domain-containing protein SgmX. SgmX, in turn, brings about polar localization of the PilB ATPase to promote T4P formation. Moreover, our data demonstrate that T4P unipolarity results from the combined action of MglA-GTP/SgmX stimulating T4P formation at the leading pole and MglB blocking this stimulation at the lagging pole by converting MglA-GTP to MglA-GDP.

## Results

### MglA Stimulates Formation of T4P.

To verify that a ∆*mglA* mutant, which contains an in-frame deletion of *mglA*, is nonmotile by means of T4P, we initially used a population-based assay. The wild-type (WT) strain displayed the long flares at the colony edge characteristic of T4P-dependent motility, while the Δ*mglA* mutant generated smooth-edged colonies similar to the Δ*pilA* mutant that lacks the major pilin PilA (*SI Appendix*, Fig. S1 *A* and *B*). Accordingly, in an assay for T4P-dependent single-cell motility, WT and a ∆*aglQ* mutant, which lacks an essential component of the gliding machinery and only moves by means of T4P (48), moved with the same speed, while the Δ*pilA* and Δ*mglA* mutants did not display motility (*SI Appendix*, Fig. S1*C*).

**Fig. 1.**
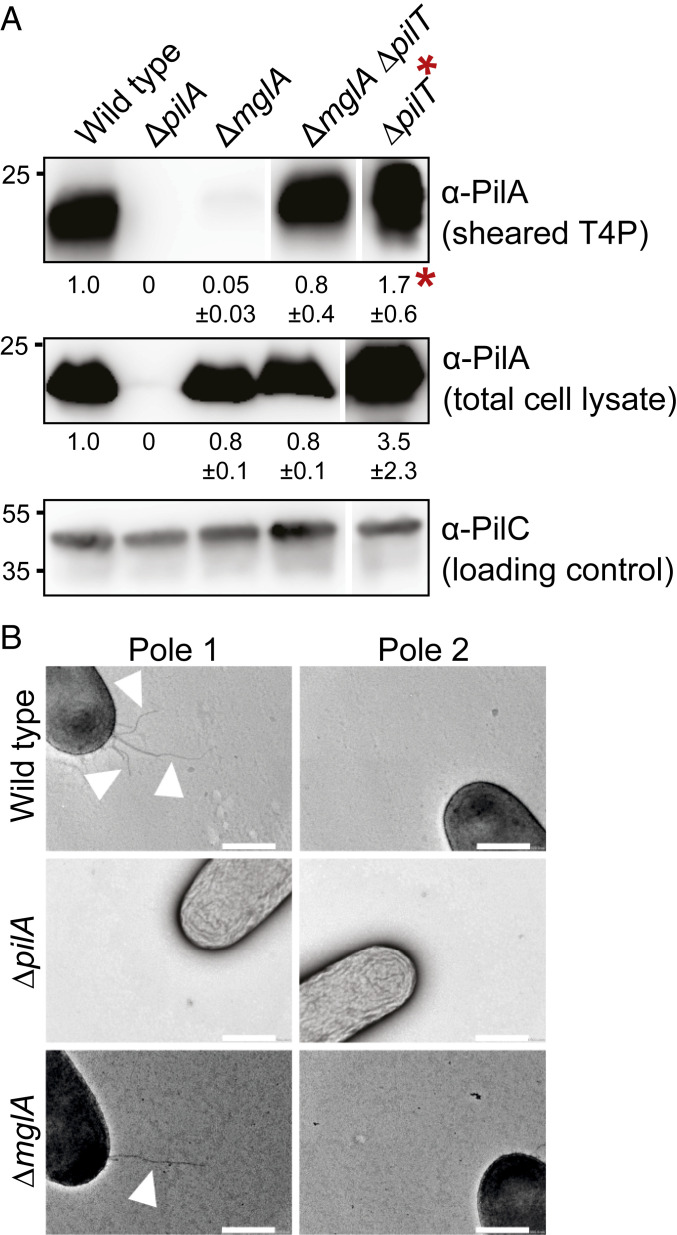
MglA stimulates T4P formation. (*A*) Immunoblot detection of PilA in sheared-off T4P and total cell lysate. (*Top*) Protein from sheared-off fraction; (*Middle* and *Bottom*) protein from total cell lysates. In all blots, protein from the same number of cells of the different strains was loaded per lane. The α-PilC blot served as a loading control. Numbers below *Top* and *Middle* indicate mean PilA signal intensity ± SD calculated from three biological replicates. To quantify PilA signals, the signal detected in the lane corresponding to ∆*pilA* mutant was subtracted from other signals. PilA signals in total cell extracts were corrected relative to the PilC loading control and normalized relative to WT (1.0) in each blot. PilA signals in the sheared fractions were used directly and normalized relative to WT (1.0). Samples in all panels are from the same blots, but lanes were removed for presentation purposes. PilA and PilC have calculated molecular masses of 23.4 and 45.2 kDa, respectively. *The sheared fraction of the ∆*pilT* mutant was diluted 200-fold before loading for comparability. (*B*) TEM analysis of T4P formation. Poles 1 and 2 represent the two poles of the same cell. Arrowheads indicate T4P. (Scale bars: 0.5 µm.)

To determine whether the ∆*mglA* mutant forms T4P, we used two methods: 1) an assay in which T4P are sheared off the cell surface followed by immunoblot quantification of PilA and 2) transmission electron microscopy (TEM). PilA was present in the sheared fraction of the ∆*mglA* mutant at a much-reduced level compared with WT, while the total cellular PilA level was similar to that in WT (Fig. 1*A*). By TEM, 31 ± 7% of ∆*mglA* cells had T4P at one pole, and the remaining cells were nonpiliated; by contrast, 83 ± 4% of WT cells had T4P at one pole, and the remainder was nonpiliated (Fig. 1*B* and Table 1). Piliated ∆*mglA* and WT cells had 1.4 ± 0.3 and 3.4 ± 0.5 T4P per piliated pole, respectively. We conclude that the ∆*mglA* mutant makes T4P but at a reduced level compared with WT.

**Table 1.** T4P localization pattern in *M. xanthus* strains

Genotype	T4P localization pattern (% of all cells)*		
	Unipolar	Bipolar	No T4P
WT	83 ± 4	0	17 ± 4
∆*pilA*	0	0	100
∆*mglA*	31 ± 7	0	69 ± 7
∆*mglB*	53 ± 3	37 ± 3	10 ± 2
*mglA*^Q82A^	47 ± 7	34 ± 11	19 ± 6
*mglB*^A64R^ ^G68R^	54 ± 8	29 ± 11	17 ± 13
∆*romR*	37 ± 25	3 ± 3	60 ± 29
∆*mglB* ∆*romR*	60 ± 9	15 ± 5	25 ± 14
∆*sgmX*	0	0	100
∆*mglA* ∆*sgmX*	0	0	100
*mglA*^Q82A^ ∆*sgmX*	0	0	100

* Three biological replicates were performed per strain with each 50 cells, and the mean ± SD was calculated.

To distinguish whether the reduced piliation level in the ∆*mglA* mutant was caused by fewer extensions or more retractions, we analyzed a ∆*mglA* ∆*pilT* double mutant, which lacks the PilT retraction ATPase. WT, the ∆*mglA* mutant, and the ∆*mglA* ∆*pilT* mutant accumulated PilA at similar levels in total cell lysates, while the level in the ∆*pilT* mutant was moderately higher (Fig. 1*A*). As expected (18), the ∆*pilT* mutant had a much higher PilA level than WT in the sheared fraction. The ∆*mglA* ∆*pilT* double mutant had a higher PilA level in the sheared fraction compared with the ∆*mglA* mutant; however, this level was still significantly lower than in the ∆*pilT* mutant (Fig. 1*A*).

The observations that the ∆*mglA* and ∆*mglA* ∆*pilT* mutants are hypopiliated compared with WT and the ∆*pilT* mutant, respectively, support that MglA is important but not essential for T4P extension. The observation that the ∆*mglA* ∆*pilT* mutant makes more T4P than the ∆*mglA* mutant supports that retractions occur in the ∆*mglA* mutant (*Discussion*).

### MglA Stimulates T4P Formation, and MglB Guarantees T4P Unipolarity.

**Fig. 2.**
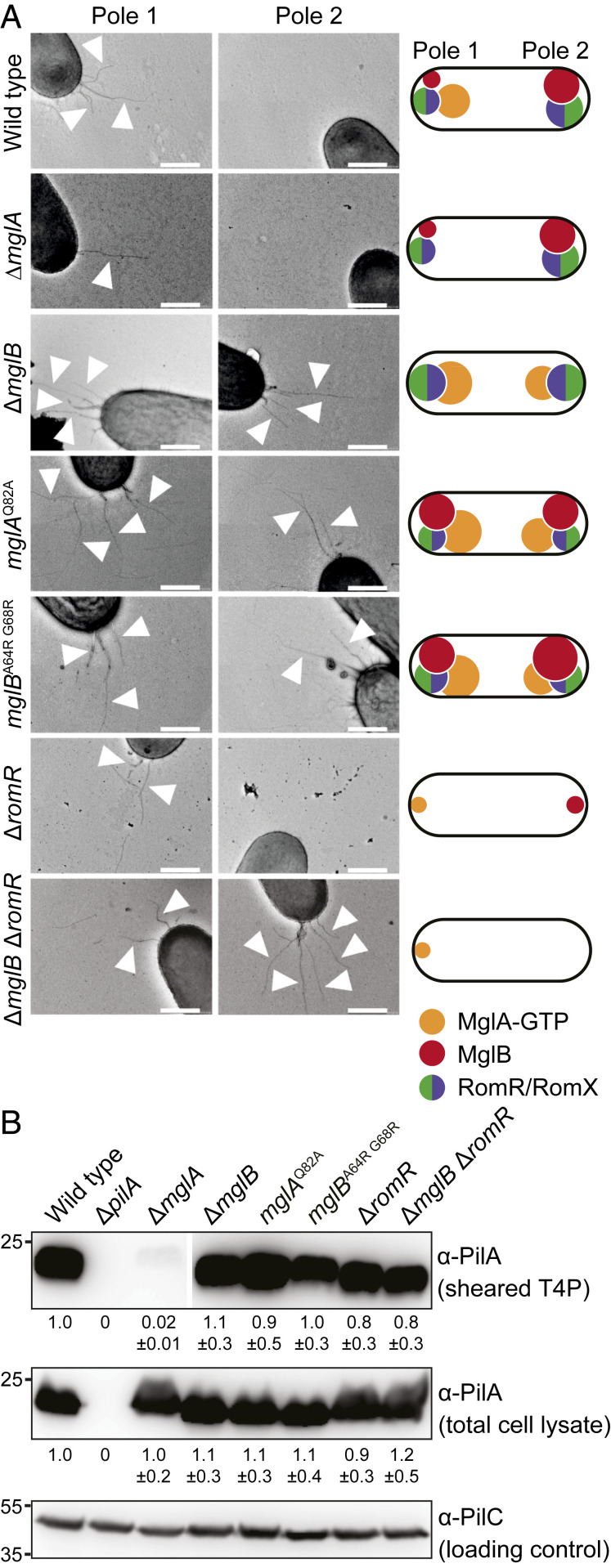
MglA-GTP is important for T4P formation. (*A*) TEM analysis of T4P formation. Poles 1 and 2 and arrowheads are the same as in Fig. 1*B*. Data for WT and ∆*mglA* mutant are the same as in Fig. 1*B*. Schematics indicate dominant localization pattern of polarity proteins in various mutants. (Scale bars: 0.5 µm.) (*B*) Immunoblot detection of PilA in sheared-off T4P and total cell lysates. Samples were prepared, and PilA signals are quantified as in Fig. 1*A* from three biological replicates. Samples in *Top* are from the same blot, but lanes were removed for presentation purposes.

We reasoned that if MglA-GTP stimulates T4P extension, then mutants in which MglA-GTP accumulates at both poles should have T4P at both poles. To this end, we analyzed T4P formation in the ∆*mglB* mutant in which polar localization of MglA-GTP is increased and shifted toward bipolar symmetric and localization of the RomR/RomX complex is shifted toward bipolar symmetric (30, 31, 41, 43, 44, 49) (Fig. 2*A* shows a schematic of average localization pattern). Based on the shear-off assay, the ∆*mglB* mutant formed T4P at a level similar to that of WT (Fig. 2*B*). Remarkably, the ∆*mglB* mutant had T4P at both poles in 37 ± 3% of cells, and 53 ± 3% had T4P at one pole (Fig. 2*A* and Table 1). These observations support that MglA-GTP stimulates T4P formation, but they did not exclude that MglB directly inhibits T4P formation at the lagging pole in WT.

To test this possibility, we analyzed T4P formation in cells containing MglA^Q82A^, a constitutively active, GTP-locked variant of MglA (31, 39, 40). In these cells, localization of MglA^Q82A^, MglB, and RomR/RomX is shifted toward bipolar symmetric (Fig. 2*A* and *SI Appendix*, Fig. S2 *A* and *D*). From the shear-off assay, the *mglA*^Q82A^ mutant formed T4P at a level similar to that of WT (Fig. 2*B*). Importantly, 34 ± 11% of *mglA*^Q82A^ cells had T4P at both poles (Fig. 2*A* and Table 1). Subsequently, we analyzed cells containing the MglB^A64RG68R^ variant, which corresponds to the *T. thermophilus* MglB^A68RA72R^ variant that has reduced GAP activity (40). In these cells, polar localization of MglA and RomR/RomX is shifted toward symmetric; MglB^A64RG68R^ localization is increased compared with MglB^WT^, but polar asymmetry is unchanged (Fig. 2*A* and *SI Appendix*, Fig. S2 *B* and *D*). From the shear-off assay, cells containing MglB^A64RG68R^ formed T4P at the same level as WT (Fig. 2*B*); however, 29 ± 11% of cells had T4P at both poles (Fig. 2*A* and Table 1). Altogether, these observations support that MglA-GTP stimulates T4P formation. Because MglB binds MglA^Q82A^ with the same affinity as MglA^WT^ but cannot stimulate MglA^Q82A^ GTPase activity (40), these observations also support that MglB in WT cells prevents T4P formation at the lagging pole by stimulating the conversion of MglA-GTP to MglA-GDP, thereby blocking MglA-GTP accumulation at this pole.

To analyze whether polar localization of MglA-GTP is important for stimulating T4P formation, we analyzed a ∆*romR* mutant. In this mutant, the MglA-GTP level is reduced (41), and polar localization of MglA and MglB strongly reduced albeit not completely abolished and both proteins localize mostly in a unipolar pattern (41, 43, 44, 49) (Fig. 2*A*). From the shear-off assay, the ∆*romR* mutant formed T4P at a level similar to that of WT (Fig. 2*B*). TEM analyses were difficult because cells were strongly adhesive, resulting in large variations in the number of cells with T4P (i.e., 37 ± 25% of cells had T4P at one pole, 3 ± 3% had T4P at both poles, and the remaining cells had no detectable T4P) (Fig. 2*A* and Table 1). These observations support that 1) the RomR/RomX GEF complex is not essential for T4P formation, 2) sufficient MglA-GTP accumulates in the ∆*romR* mutant to stimulate T4P formation, and 3) polar localization of MglA-GTP may not be important for T4P formation. More importantly, they support that cells in which MglA and MglB localize to opposite poles are almost exclusively unipolarly piliated. In agreement with these observations, in a ∆*mglB* ∆*romR* mutant, in which MglA polar localization is strongly reduced and mostly unipolar (Fig. 2*A*) (49), 15 ± 5% of cells had T4P at both poles (Fig. 2 and Table 1).

Collectively, these observations support that T4P unipolarity in WT cells is the result of MglA-GTP stimulation of T4P formation at the leading pole and MglB blocking this stimulation at the lagging pole by promoting MglA-GTP conversion to MglA-GDP.

### T4P Are Active at Both Poles in Bipolarly Piliated Cells.

To test whether T4P in bipolarly piliated cells are active at both poles, we tracked single cells. To ensure that cells only move by means of T4P, strains contained the ∆*aglQ* mutation. To distinguish between Frz-dependent and -independent reversals, a set of strains also contained a ∆*frzE* mutation. In all strains, the same fraction of cells displayed movement, and all strains moved with similar speed (*SI Appendix*, Fig. S3*A*). However, cells lacking MglB or containing MglA^Q82A^ or MglB^A64RG68R^ reversed at a significantly higher frequency than the ∆*aglQ* cells in the presence as well as in the absence of the FrzE kinase (*SI Appendix*, Fig. S3*B*). We confirmed that cells of the ∆*romR* mutant reversed at a much-reduced frequency compared with WT (41, 45). Consistently, calculation of the mean squared displacement (MSD) showed clear differences between mutants (*SI Appendix*, Fig. S3*C*). The MSD for ∆*aglQ* cells followed a linear trend consistent with a random walk, MSD of the ∆*romR* mutant had an increasing slope consistent with motion without reversals, and MSD for mutants with many bipolarly piliated cells reached a plateau consistent with little net displacement.

We take the observation that the mutants that are bipolarly piliated reverse in an Frz-independent manner as support that T4P are active at both poles (*Discussion*). The ∆*romR* mutant displayed the same fraction of moving cells as the other strains analyzed, supporting that this mutant makes T4P at the same level at the other strains as suggested in the shear-off assay (Fig. 2*B*). These results demonstrate that MglA-GTP stimulates the formation of active T4P. Moreover, we conclude that neither MglB nor RomR, and by implication, also not RomX, are required for T4P function. Rather, MglB is important for establishing T4P unipolarity, and RomR is important for reversals.

### Lack of MglA Affects Neither Accumulation of T4PM Proteins nor T4PM Assembly.

**Fig. 3.**
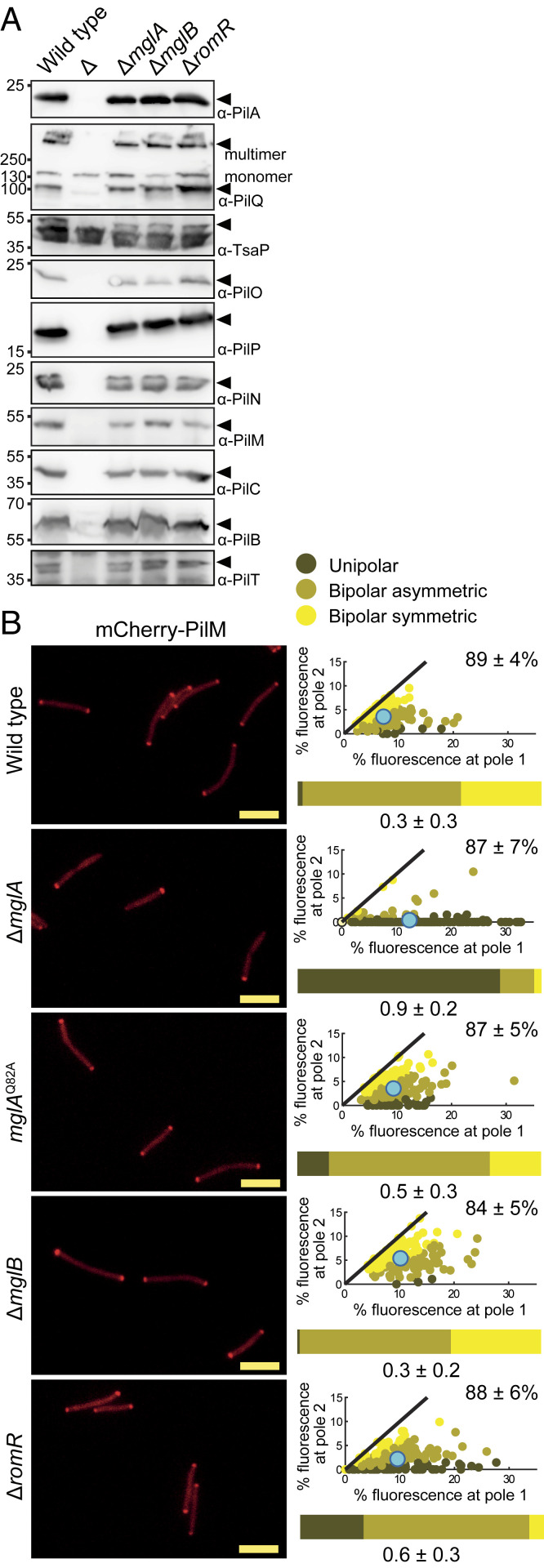
Cells lacking MglA assemble T4PM. (*A*) Immunoblot analysis of accumulation of core T4PM proteins. Total cell lysates were loaded from the same number of cells per lane. Analyzed proteins are indicated on the right; ∆ indicates cell lysate loaded from the relevant in-frame deletion mutant. In the PilQ blot, the upper band corresponds to the heat- and detergent-resistant PilQ multimer, while the lower band corresponds to monomers. (*B*) Localization of mCherry-PilM by epifluorescence microscopy. For each cell with polar clusters, an asymmetry index (*ω*) was calculated (*Materials and Methods*) to distinguish between unipolar, asymmetric bipolar, and symmetric bipolar localization; cells with no polar signal were categorized as having diffuse localization and have an *ω* = 0. In the scatterplots, the percentage of total fluorescence at pole 2 is plotted against the percentage of total fluorescence at pole 1 for all cells with polar cluster(s), and individual cells are color coded according to its localization pattern. Pole 1 is per definition the pole with the highest fluorescence. Black lines are symmetry lines, cyan dots show the mean, and numbers in upper right corners are the mean percentage of total fluorescence ± SD in the cytoplasm. Horizontal bars show the percentage of cells with the indicated localization patterns according to the color code. Numbers below indicate the mean *ω* ± SD; *N* = 150 cells for all strains. The data for WT are the same as in Fig. 7. (Scale bars: 5 µm.)

To elucidate how MglA-GTP stimulates T4P formation, we examined by immunoblot analysis the accumulation of the 10 core T4PM proteins. The ∆*mglA* mutant accumulated these 10 proteins similarly to WT (Fig. 3*A*). The ∆*mglB* and ∆*romR* mutants also accumulated the core proteins at WT levels (Fig. 3*A*), excluding that the abnormal piliation pattern in the ∆*mglB* mutant and the reduced reversals in the ∆*romR* mutant were related to accumulation of the core proteins.

Next, we asked whether T4PM assembles in the ∆*mglA* mutant. In *M. xanthus*, T4PM assembles at both cell poles in an outside-in manner starting with the PilQ secretin in the outer membrane (10, 36). Because the bipolar localization of the cytoplasmic PilM protein is a readout of its incorporation into the T4PM and depends on PilQ, PilP, PilO, and PilN (36), we used polar localization of an active mCherry-PilM fusion as a proxy for T4PM assembly (*SI Appendix*, Fig. S4 *A*–*C*). As previously shown (35), mCherry-PilM mostly localized bipolarly in WT; however, in the ∆*mglA* mutant, this pattern was shifted toward unipolar (Fig. 3*B*). In the presence of the GTP-locked MglA^Q82A^ variant as well as in the absence of MglB or RomR, mCherry-PilM localized essentially as in WT (Fig. 3*B*), supporting that T4PM is assembled at both poles in these mutants. Consistent with these localization patterns, we observed that an active PilQ-sfGFP fusion expressed from the native site (*SI Appendix*, Fig. S4 *D* and *E*) was mostly bipolar in WT as previously observed (36, 37) but shifted toward unipolar in the ∆*mglA* mutant (*SI Appendix*, Fig. S5).

We conclude that MglA, as previously described for PilB and PilT (30, 50), is not required for polar localization of PilQ and PilM and infer that the T4PM assembles in the absence of MglA. Because PilQ and PilM are shifted toward unipolar in the ∆*mglA* mutant, these observations also suggest that T4PM mostly assembles at one pole in this mutant. These observations, therefore, suggest that the ∆*mglA* mutant is unipolarly piliated because T4PM only assembles at one pole; by contrast, WT assembles T4PM at both poles but is unipolarly piliated because MglA-GTP can only stimulate T4P formation at one pole.

### MglA-GTP Interacts with SgmX In Vitro.

We hypothesized that MglA-GTP interacts directly with cytoplasmic components of the T4PM to stimulate T4P formation. To this end, we used the bacterial adenylate cyclase two-hybrid system (51) and generated C- and T-terminal fusions with the T18 and T25 fragments to the three cytoplasmic proteins of T4PM (PilM, PilB, PilT) (*SI Appendix*, Fig. S1*A*) and MglA. In control experiments, we observed the expected interactions; however, we did not detect interactions between MglA and PilB, PilT, or PilM, implying that additional factor(s) could be involved in connecting MglA and T4PM.

In *B. bacteriovorus*, MglA is important for T4P formation and predation and interacts directly with the TPR domain-containing protein Bd2492, which is also important for predation (26), while its function in T4P function is unknown. Because SgmX (MXAN_5766) (*SI Appendix*, Fig. S7*A*) is a homolog of Bd2492 (26) and a transposon insertion in *sgmX* causes a defect in T4P-dependent motility (52), we hypothesized that SgmX could link MglA and T4PM in *M. xanthus*.

**Fig. 4.**
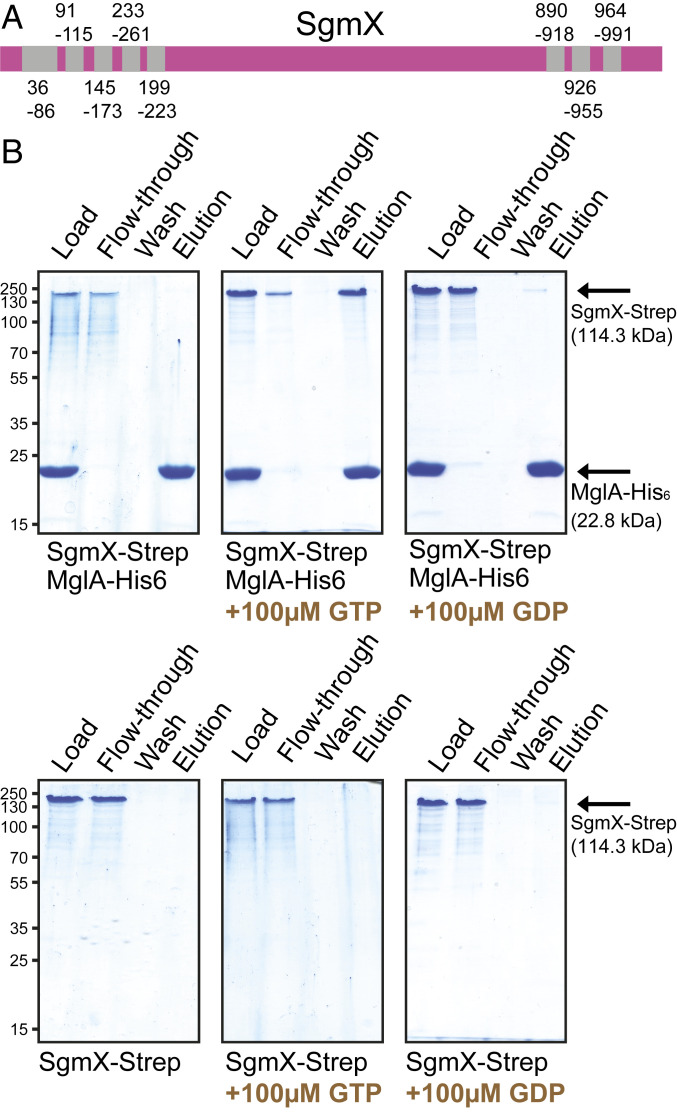
SgmX interacts with MglA-GTP in vitro. (*A*) Domain structure of SgmX. Coordinates of TPR domains (gray) are indicated. (*B*) SgmX-Strep and MglA-His_6_-GTP interact. MglA-His_6_ was preloaded with nucleotide as indicated prior to addition of SgmX-Strep. Proteins were applied to a nickel-nitrilotriacetic acid (Ni^2+^-NTA)–agarose resin, the resin was washed, and bound protein was eluted with 400 mM imidazole. Fractions before and after loading, the last wash, and the elution fraction were separated by sodium dodecyl sulfate–polyacrylamide gel electrophoresis (SDS/PAGE), and gels were stained with Coomassie Brilliant Blue. Equivalent volumes of the load, flow through, and wash and threefold more of the elution fraction were loaded. For each protein combination, fractions were separated on the same gel. Calculated molecular masses of monomeric MglA-His_6_ and SgmX-Strep are indicated. Note that purified SgmX-Strep forms a heat- and detergent-resistant dimer.

SgmX is a 1,060-amino acid protein with five and three TPR domains in the N terminus and C terminus, respectively (Fig. 4*A*). Generally, TPR domains are involved in protein–protein interactions (53). The region between the TPR domains does not match known protein domains. Sequence analyses support that SgmX contains neither a signal peptide nor transmembrane domains and is likely cytoplasmic similarly to Bd2492 (26).

To determine whether MglA and SgmX interact, we performed pull-down experiments in vitro using purified proteins (*SI Appendix*, Fig. S8). A C-terminal Strep-tagged SgmX variant had a molecular mass of ∼233 kDa in SDS/PAGE, suggesting that SgmX is a stable dimer (*SI Appendix*, Fig. S8). For the pull-down experiments, MglA-His_6_ was preincubated with GDP, GTP, or no nucleotide and then mixed with SgmX-Strep. SgmX-Strep was retained on an Ni^2+^-NTA resin in the presence of MglA-His_6_-GTP but not in the presence of MglA-His_6_-GDP or MglA-His_6_ without a preloaded nucleotide (Fig. 4*B*). In control experiments, SgmX-Strep alone did not bind the Ni^2+^-NTA resin. We conclude that SgmX interacts with MglA-GTP but not with MglA-GDP, consistent with SgmX being an effector of MglA-GTP.

### SgmX Acts Together with MglA to Stimulate T4P Formation.

**Fig. 5.**
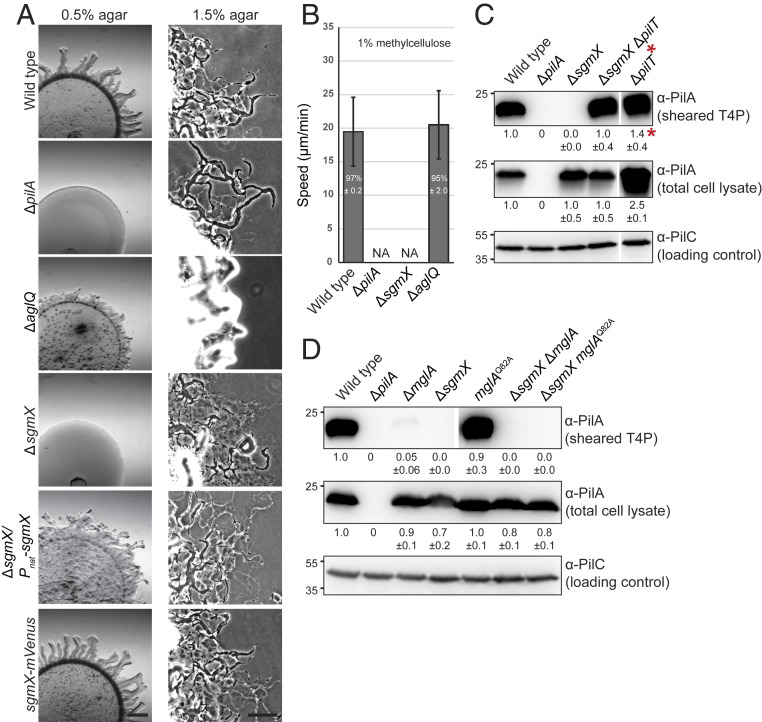
SgmX is important for T4P formation. (*A*) SgmX is essential for T4P-dependent motility. T4P-dependent motility was analyzed as in *SI Appendix*, Fig. S1*B*. For gliding motility, cells were incubated on 1.5% agar and 0.5% CTT. *sgmX* was expressed from the native *sgmX* promoter (*SI Appendix*, Fig. S7*B*) from a plasmid integrated in a single copy at the Mx8 *attB* site. (Scale bars: *Left*, 1 mm; *Right*, 100 µm.) (*B*) Speed of single cells moving by T4P-dependent motility. For the WT, ∆*pilA*, and ∆*aglQ* strains, cells analyzed are the same as in *SI Appendix*, Fig. S1*C*. *N* = 20 cells for each strain from three independent experiments. NA, not applicable. (*C* and *D*) Immunoblot detection of PilA in sheared-off T4P and total cell lysates. Samples were prepared and analyzed as in Fig. 1*A*. PilA signals were quantified as in Fig. 1*A* from three different biological replicates for each blot. All samples in a panel are from the same blot, but lanes were removed for presentation purposes. *Sheared fraction of ∆*pilT* mutant was diluted 200-fold before loading for comparability (*C*).

A Δ*sgmX* mutant formed colonies with a smooth edge similar to the Δ*pilA* mutant on 0.5% agar, which is favorable to T4P-dependent motility (Fig. 5*A*). On 1.5% agar, which is favorable to gliding motility, WT, ∆*pilA* mutant, and ∆*sgmX* mutant displayed single cells at the colony edge characteristic of gliding, while the Δ*aglQ* mutant did not. In single-cell assays for T4P-dependent motility, Δ*sgmX* cells did not display movement (Fig. 5*B*). The motility defect of the Δ*sgmX* mutant was complemented by ectopically expressed *sgmX* as well as by an SgmX-mVenus fusion protein synthesized from the native *sgmX* locus (Fig. 5*A* and *SI Appendix*, Figs. S7*B* and S9*A*). We conclude that SgmX is essential for T4P-dependent motility but not for gliding.

From the shear-off assay, the ∆*sgmX* mutant did not detectably form T4P, while PilA in the total cell fraction was similar to that in WT (Fig. 5*C*). Consistently, T4P were not detected by TEM in this mutant (Table 1). From the shear-off assay, the ∆*sgmX* ∆*pilT* double mutant formed T4P; however, the PilA level in the sheared fraction was significantly lower than in the ∆*pilT* mutant (Fig. 5*C*). We conclude that SgmX is important but not essential for T4P extension. Similar to the ∆*mglA* ∆*pilT* mutant, the observation that the ∆*sgmX* ∆*pilT* mutant makes more T4P than the ∆*sgmX* mutant also supports that retractions occur in this mutant (*Discussion*).

T4P formation was detectable neither in the *∆sgmX* ∆*mglA* mutant nor in the ∆*sgmX mglA*^Q82A^ mutant (Fig. 5*D* and Table 1). The SgmX-mVenus fusion accumulated in the absence of MglA and in the presence of MglA^Q82A^ (*SI Appendix*, Fig. S9*A*), and MgA^WT^ and MglA^Q82A^ accumulated in the absence of SgmX (*SI Appendix*, Fig. S9 *B* and *C*). Because the ∆*mglA* mutant forms a low but detectable level of T4P (Figs. 1*A*, 2*B*, and 5*D* and Table 1) and T4P formation in the ∆*sgmX* mutant is undetectable (Fig. 5 *C* and *D* and Table 1), these results support that *mglA* and *sgmX* act in the same genetic pathway and are consistent with SgmX acting downstream of MglA to stimulate T4P formation and SgmX being an effector of MglA-GTP that functions between MglA-GTP and T4P formation.

### SgmX Localizes Dynamically to the Leading Cell Pole.

**Fig. 6.**
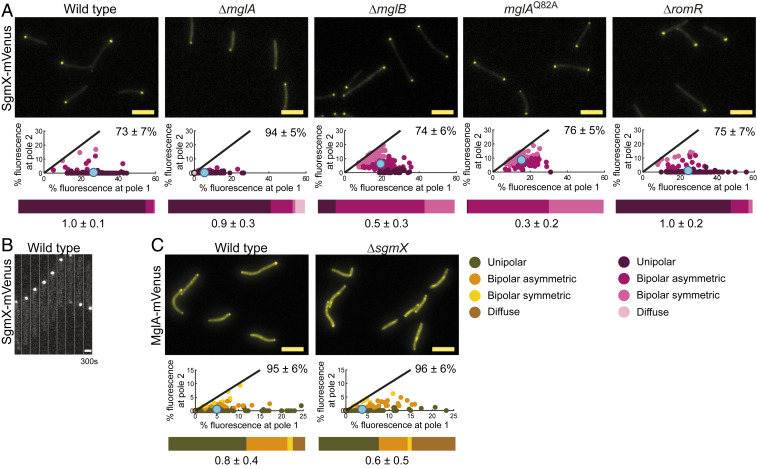
Polar SgmX localization is enhanced by MglA. (*A* and *C*) Localization of SgmX-mVenus and MglA-mVenus. Cells were treated and analyzed as in Fig. 3*B*. *N* = 150 cells for each strain. For MglA-mVenus in WT, data are the same as in *SI Appendix*, Fig. S2*B*. (Scale bars: 5 µm.) (*B*) SgmX-mVenus is dynamically localized to the leading pole. Cells were imaged by time-lapse fluorescence microscopy at 30-s intervals for 300 s. (Scale bar: 1 µm.)

We determined the localization of SgmX and observed that the active SgmX-mVenus fusion localized in a unipolar pattern in the vast majority of cells in snapshots (Fig. 6*A*). Time-lapse microscopy demonstrated that SgmX-mVenus localized to the leading cell pole in moving cells and switched polarity during reversals (Fig. 6*B*).

Next, we determined whether MglA or its regulators are important for polar SgmX localization (Fig. 6*A* and *SI Appendix*, Fig. S9*A*). In the absence of MglA, total polar localization of SgmX-mVenus was significantly reduced compared with WT, but polar asymmetry was unchanged. In the absence of MglB or in the presence of MglA^Q82A^ (in which MglA-GTP is shifted toward symmetric bipolar) (Fig. 2*A*), SgmX-mVenus localization was shifted toward bipolar without affecting the total polar signal. In the ∆*romR* mutant, in which polar localization of MglA-GTP is reduced but not abolished (Fig. 2*A*), the polar SgmX-mVenus signal and asymmetry were similar to those in WT. These observations support that MglA-GTP stimulates but is not essential for polar SgmX localization, suggesting that other factors are involved in bringing about polar SgmX localization.

To test whether SgmX is important for MglA localization, we used an endogenous MglA-mVenus fusion (*SI Appendix*, Fig. S9*B*). MglA-mVenus localized mostly unipolarly or bipolar asymmetrically in WT; in the absence of SgmX, the polar MglA-mVenus signal was slightly reduced, but most cells still had the unipolar to bipolar asymmetric localization pattern (Fig. 6*C*). Lack of SgmX affected neither the total polar signals nor asymmetry of MglB-mCherry but resulted in more asymmetric RomR-mCherry localization (*SI Appendix*, Fig. S10). Overall, these observations suggest that SgmX may have a minor role in bringing about polar localization of MglA-GTP, thus supporting the interaction between SgmX and MglA-GTP.

### SgmX Stimulates Polar Localization of the PilB Extension ATPase.

**Fig. 7.**
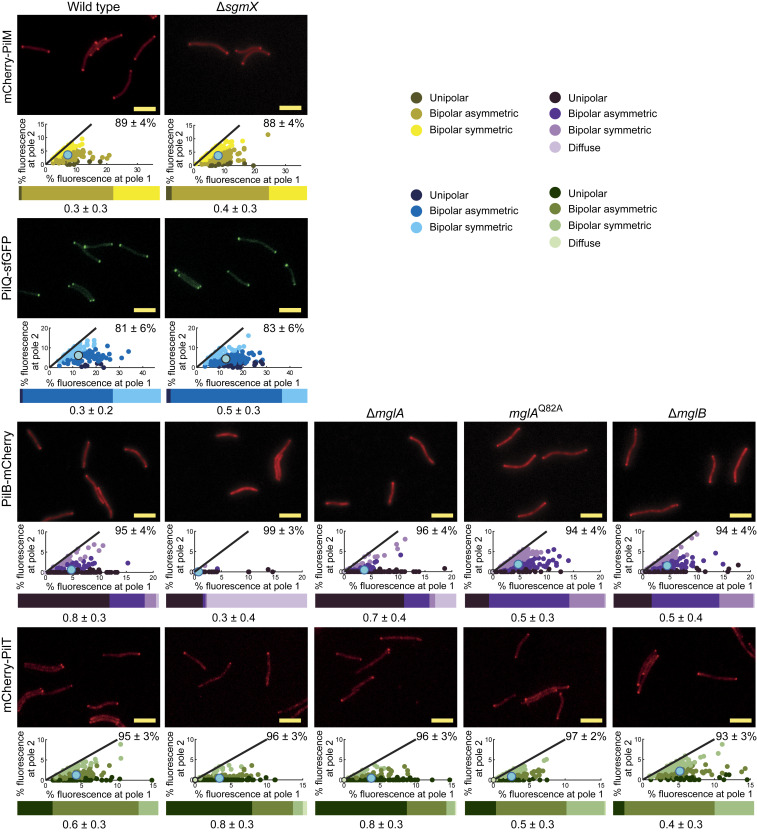
SgmX stimulates polar localization of PilB. Localization of mCherry-PilM, PilQ-sfGFP, PilB-mCherry, and mCherry-PilT. Cells were treated and analyzed as in Fig. 3*B*. The data for mCherry-PilM and PilQ-sfGFP in WT are the same as in Fig. 3*B* and *SI Appendix*, Fig. S5, respectively. *N* = 150 cells for each strain. (Scale bars: 5 µm.)

In the ∆*sgmX* mutant, the 10 core T4PM proteins accumulated as in WT (*SI Appendix*, Fig. S11), and PilQ-sfGFP and mCherry-PilM localized as in WT (Fig. 7 and *SI Appendix*, Fig. S4 *C* and *E*), supporting that the T4PM assembles at both poles in the absence of SgmX. Importantly, polar localization of PilB-mCherry was almost completely abolished in the ∆*sgmX* mutant, while polar localization of mCherry-PilT was similar to that in WT except that it localized more unipolarly (Fig. 7 and *SI Appendix*, Fig. S12). We confirmed that PilB-mCherry and mCherry-PilT were mostly unipolarly localized in the absence of MglA (Fig. 7). Moreover, in the *∆sgmX* ∆*mglA* double mutant, PilB-mCherry and mCherry-PilT localized as in the *∆sgmX* mutant (*SI Appendix*, Fig. S13). Thus, SgmX is important for polar localization of PilB, supporting a model whereby SgmX could stimulate T4P formation by stimulating, directly or indirectly, polar localization of PilB. Consistently, we observed that in the presence of the GTP-locked MglA^Q82A^ variant and in the ∆*mglB* mutant, PilB-mCherry localization shifted toward bipolar (Fig. 7), while mCherry-PilT was slightly more symmetric (Fig. 7).

## Discussion

Here, we addressed how the small GTPase MglA stimulates T4P-dependent motility in *M. xanthus*. We report that MglA-GTP stimulates T4P formation. We identify the TPR domain-containing protein SgmX as an effector that interacts directly with MglA-GTP and provide in vivo evidence supporting that SgmX acts downstream of MglA-GTP to stimulate T4P formation, possibly by direct or indirect interaction with the PilB extension ATPase. Moreover, we provide evidence that MglB, the MglA GAP, generates T4P unipolarity by excluding MglA-GTP from the lagging pole. Thus, the unipolar T4P pattern is brought about by the stimulatory effect of MglA-GTP together with SgmX on T4P formation at the leading pole and the inhibitory effect of MglB on MglA-GTP at the lagging pole.

Several lines of evidence suggest that MglA-GTP and SgmX stimulate T4P extension. First, mutants lacking only MglA or only SgmX made fewer T4P compared with WT or no T4P, respectively, but still accumulated the core T4PM proteins and assembled T4PM. Second, double mutants lacking MglA and the PilT retraction ATPase or SgmX and PilT both made fewer T4P than cells lacking only PilT. Third, in mutants with MglA-GTP and SgmX localization shifted toward bipolar, a large fraction of cells had T4P at both poles. The effect of MglA-GTP and SgmX on T4P retraction is less clear. Mutants lacking MglA and PilT or SgmX and PilT made more T4P than mutants lacking only MglA or SgmX, respectively, supporting that retractions occur in cells lacking MglA or SgmX. However, these cells are nonmotile by means of T4P, suggesting that these retractions are infrequent and that the two proteins are important for retractions. Alternatively, T4P retraction occurs independently of MglA and SgmX, but cells make too few T4P to generate cell movement. To clarify the function of MglA and SgmX in retractions, their effect on retractions of individual T4P will have to be determined in future experiments.

Several lines of evidence support that SgmX is an effector of MglA-GTP and acts downstream of MglA-GTP to stimulate T4P formation. Firstly, epistasis experiments using a ∆*sgmX* mutation together with *mglA* loss-of-function and gain-of-function mutations are consistent with SgmX acting downstream of MglA-GTP to stimulate T4P formation. Secondly, SgmX interacts directly with MglA-GTP but not with MglA-GDP in vitro. Thirdly, SgmX is unipolarly localized to the leading pole in WT, and MglA is important for this localization. Consistently, in mutants with MglA localization shifted toward bipolar, SgmX was also more bipolar, and these cells were bipolarly piliated. Finally, SgmX stimulated polar localization of the PilB extension ATPase in otherwise WT cells, and in cells with bipolar SgmX, PilB localization was shifted toward bipolar. Collectively, these findings support a model in which MglA-GTP directly interacts with SgmX, allowing SgmX to efficiently support T4P formation. The observations that cells lacking only SgmX do not detectably form T4P, while cells lacking only MglA do, support that SgmX independently of MglA can somewhat stimulate T4P formation. These observations also support that MglA-GTP may not only stimulate polar localization of SgmX but also bring about a conformational change in SgmX that results in activation of SgmX. We speculate that stimulation of T4P formation by SgmX may involve a direct or indirect interaction with PilB because SgmX is important for polar localization of PilB. In *P. aeruginosa*, the FimX/c-di-GMP complex localizes to the piliated pole and interacts directly with PilB to stimulate PilB polar localization and T4P extension (19). Thus, MglA-GTP/SgmX complex may function analogously to FimX/c-di-GMP in *P. aeruginosa*.

Similarly, to the rod-shaped cells of *P. aeruginosa* (20) and *T. thermophilus* (27), *M. xanthus* cells are unipolarly piliated. In *M. xanthus,* however, the pole at which T4P are formed switches rapidly during reversals. Thus, both poles are competent for T4P extension/retraction, but only one pole at a time is licensed to extend T4P. Our data demonstrate that MglB is essential for this T4P unipolarity. Specifically, our data support that MglB via its GAP activity prevents accumulation of MglA-GTP at the lagging pole and therefore, stimulation of T4P formation via SgmX. Thus, T4P unipolarity in *M. xanthus* is the result of stimulation of T4P formation by MglA-GTP together with SgmX at the leading pole in combination with MglB GAP activity at the lagging pole inhibiting MglA-GTP and SgmX accumulation at this pole. How unipolarity of T4P is brought about in *P. aeruginosa* and *T. thermophilus* is not known except that FimX/c-di-GMP in *P. aeruginosa* is essential for unipolar T4P formation at low c-di-GMP concentrations, while lack of FimX in combination with high c-di-GMP concentrations by an unknown mechanism cause formation of T4P along the cell body (19, 20), and in *T. thermophilus*, lack of MglA, MglB, or both results in T4P formation at both poles (27).

Coccoid *Neisseria gonorrhoeae* are peritrichously piliated but still display net movement by means of T4P. This observation was rationalized as a tug-of-war phenomenon in which juxtaposed T4P form bundles on “one side” of the spherical cells to generate net movement (54). Our data support that T4P in bipolarly piliated *M. xanthus* cells are active because these cells hyperreverse independently of the Frz system. We interpret these hyperreversals as the result of a tug-of-war between T4P at opposite cell poles, and the pole that “wins” becomes the leading pole until retractions become more dominant at the opposite pole, forcing a change in direction of movement. However, bipolarly piliated *M. xanthus* cells display little net movement, supporting that piliation patterns are tailored to the needs of individual species.

Interestingly, the regulators of T4P formation and localization in *P. aeruginosa* and *X. axonopodis* pv. citri center on the second messenger c-di-GMP. Similarly, in the gamma-proteobacterium *Vibrio cholerae*, c-di-GMP directly binds to the PilB ortholog MshE to stimulate T4P extension (55, 56), and in *Clostridium perfringens*, binding of c-di-GMP to the PilB ortholog PilB2 was suggested to stimulate T4P extension (57). By contrast, in *M. xanthus*, MglA together with SgmX and MglB regulates T4P formation and localization independently of c-di-GMP and responds to Frz signaling by switching the pole at which T4P are formed.

The MglA/MglB/RomR/RomX module also regulates gliding motility. MglA-GTP stimulates assembly of the Agl/Glt gliding motility complexes at the leading pole and is incorporated into these complexes (38, 39). The Agl/Glt complexes disassemble as they reach the lagging cell pole because they are sensitive to MglB at this pole (39). In the absence of MglB or in the presence of MglA^Q82A^, gliding cells hyperreverse independently of the Frz system and with little net movement (39). Thus, for both motility systems, MglB at the lagging pole is important for persistent directional motion without Frz-independent reversals. Altogether, the spatial arrangement of MglA-GTP, MglB, RomR/RomX, and SgmX guarantees the assembly of both motility machineries at the leading pole, that T4P are not formed at the lagging pole, and that the gliding motility complexes disassemble as they reach this pole.

The data presented here support that MglA-GTP stimulates T4P formation via SgmX. However, MglA may have an additional function independent of SgmX. In the absence of MglA, the localization of PilQ and PilM is shifted toward unipolar. We previously showed that PilB and PilT localize to the same pole in the absence of MglA (30, 50). These observations support that all T4PM proteins may localize to the same pole and that T4PM assembles at one pole in the absence of MglA. Overall, this would explain why cells lacking MglA are unipolarly piliated. Because T4PM assembly initiates with PilQ in the outer membrane at the new pole during cell division (36), these observations suggest that MglA-GTP may stimulate PilQ incorporation at the new pole. How this effect of MglA is implemented remains to be investigated.

## Materials and Methods

### Cell Growth and Construction of Strains.

DK1622 was used as WT *M. xanthus* (58) throughout, and all strains are derivatives of DK1622. *M. xanthus* strains used are listed in *SI Appendix*, Table S1; plasmids are in *SI Appendix*, Table S2; and primers are in *SI Appendix*, Table S3. *M. xanthus* was grown at 32 °C in 1% CTT broth (59) or on 1.5% agar supplemented with 1% CTT and kanamycin (50 µg/mL) or oxytetracycline (10 µg/mL) as appropriate. In-frame deletions were generated as described in ref. 60. For ectopic gene expression, plasmids were integrated at the Mx8 *attB* site.

### Microscopy and Analysis of Fluorescence Microscopy Images.

For phase contrast and fluorescence microscopy, cells were treated, and images were recorded and analyzed as described in ref. 41. Briefly, exponentially growing cells were placed on a thin 1.5% agarose pad buffered with 10 mM Tris⋅HCl, pH 8.0, 1 mM potassium phosphate buffer, pH 7.6, and 8 mM MgSO_4_ supplemented with 0.2% CTT on a glass slide; covered with a coverslip; incubated for 30 min at 32 °C; and visualized at 32 °C using a temperature-controlled DM6000B microscope (Leica) with a Plan Apochromat 100×/NA 1.40 oil objective (Leica) and a Cascade II 1024 camera (Roper Scientific). Cells in phase contrast images were automatically detected using Oufti (Paintdakhi). Fluorescence signals in segmented cells were identified and analyzed using a custom-made Matlab v2016b (MathWorks) script that divides a cell into polar region 1, polar region 2, and the cytoplasmic region. Polar regions are defined as the regions of a cell within a distance of 10 pixels, corresponding to 0.64 µm, from a tip of the cell. The cytoplasmic region includes all pixels of the cell with the exception of the polar regions. A polar cluster was identified when three or more connected pixels within a polar region had a fluorescence signal higher than a cell-specific threshold signal of two SDs above the average fluorescence signal in the cytoplasmic region. The fluorescence of a polar cluster was defined as the sum of the fluorescence signal of all connected pixels that exceeded the threshold value in that polar region. The cytoplasmic signal was defined as the sum of the fluorescence signal of all pixels between the two polar regions. For each cell with polar cluster(s), an asymmetry index (*ω*) was calculated as

$$\omega =\frac{total\;fluorescence\;at\;pole\;1-total\;fluorescence\;at\;pole\;2}{total\;fluorescence\;at\;pole\;1+total\;fluorescence\;at\;pole\;2}.$$

By definition, pole 1 is the pole with the highest fluorescence. *ω* varies between zero (bipolar symmetric localization) and one (unipolar localization). The localization patterns were binned into three categories as follows: unipolar (*ω* > 0.9), bipolar asymmetric (0.9 > *ω* > 0.2), and bipolar symmetric (*ω* < 0.2). Diffuse localization was determined when no polar signal was detected, and for these cells, *ω* was set to zero. For time-lapse epifluorescence microscopy, cells were prepared as described and recorded for 15 min with images captured every 30 s. Data were processed with Metamorph 7.5 (Molecular Devices) and ImageJ 1.52b (61).

## Supplementary Material

Supplementary File

## Data Availability

All data supporting this study are available within the article and *SI Appendix*.
