# Supplementary material for: The small GTPase MglA together with the TPR domain protein SgmX stimulates type IV pili formation in M. xanthus
Source: Proc Natl Acad Sci U S A. 2020 Sep 8;117(38):23859–68. doi: 10.1073/pnas.2004722117 (PMC7519303; doi:10.1073/pnas.2004722117)
Supplement: Supplementary File [file pnas.2004722117.sapp.pdf]

PNAS
www.pnas.org

Supplementary Information

The small GTPase MglA together with the TPR domain protein SgmX stimulate type IV pili formation in *M. xanthus*

Anna Potapova, Luís Antonío Menezes Carreira & Lotte Søgaard-Andersen

Corresponding author: Lotte Søgaard-Andersen[1]
E-mail: sogaard@mpi-marburg.mpg.de

This PDF file includes:

Supplementary text
Figures S1 to S13
Tables S1 to S3
SI References

www.pnas.org/cgi/doi/10.1073/pnas.2004722117

Supplementary text:
Supplementary Materials & Methods

Cell growth and construction of strains. All in-frame deletions and plasmid integrations were verified by PCR. Plasmids were propagated in *Escherichia coli* Mach1 [Δ*recA1398 endA1 tonA* φ80Δ*lacZ*ΔM15 Δ*lacX74* hsdR(r_K^- m_K^+)]. *E. coli* cells were grown in LB or on 1.5% agar plates containing LB at 37°C with added antibiotics if appropriate (1). All DNA fragments generated by PCR were verified by sequencing.

Motility assays and determination of reversal frequency. Motility assays were done according to (2). Briefly, in population-based assays, *M. xanthus* cells from exponentially growing cultures were harvested at 4000 *g* for 10 min at room temperature (RT) and resuspended in 1% CTT to a calculated density of 7×10^9 cells/ml. 5 µl aliquots of cell suspensions were placed on 0.5% agar plates supplemented with 0.5% CTT for T4P-dependent motility and 1.5% agar plates supplemented with 0.5% CTT for gliding motility (3) and incubated at 32°C. After 24 hrs, colony edges were visualized using a Leica M205FA stereomicroscope and imaged using a Hamamatsu ORCA-flash V2 Digital CMOS camera (Hamamatsu Photonics). For higher magnifications of cells at colony edges on 1.5% agar, cells were visualized using a Leica DM6000B microscope and imaged using a Cascade II 1024 EMCCD camera (Photometrics). To track individual cells moving by T4P-dependent motility, 5 µl of exponentially growing cultures were spotted into a 24-well polystyrene plate (Falcon). After 10 min at RT, cells were covered with 500 µl of 1% methylcellulose in MMC buffer (10 mM MOPS, 4 mM $MgSO_4$, 2 mM $CaCl_2$, pH 7.6), and incubated at RT for 30 min. Subsequently, cells were visualized for 10 min at 20 s intervals at RT using a Leica DMi8 inverted microscope and imaged using a Leica DFC9000 GT camera. Individual cells were tracked using Metamorph 7.5 (Molecular Devices) and ImageJ 1.52b (4). 20 cells were tracked per strain per biological replicate. For each cell, the distance moved per 20 s interval was determined and the mean speed calculated as µm/min. For reversals, 50 cells were tracked as described and the number of reversals per cell per 10 min determined manually. To calculate mean squared displacement (MSD) of cells, the distance of a cell to its original position at t=0 was determined for every time interval of a time-lapse series, squared and plotted as function of time. Cells were followed for 10 min and recorded every 20 s. For each strain 50 cells were followed.

Bacterial two-hybrid assay. Plasmids listed in Table S2 were transformed pairwise into chemically competent *E. coli* BTH101 cells [F^- *cya-99 araD139 galE15 galK16 rpsL1* (Str^r) *hsdR2 mcrA1 mcrB1*] (Euromedex, France), and cells plated on LB plates containing 50 µg/ml ampicillin, 30 µg/ml kanamycin, 1 mM isopropyl-β-D-thiogalactopyranoside (IPTG), and 40 µg/ml 5-bromo-4-chloro-3-indolyl-β-D-galactopyranoside (X-gal). Plates were incubated at 30°C for 48 hrs, and then randomly selected individual colonies were suspended in 50 µl LB, and 5 µl were spotted onto fresh LB ampicillin/kanamycin/IPTG/X-gal plates and incubated for 48 hrs at 30°C and imaged using an Epson Perfection V700 Photo scanner.

Transmission electron microscopy. Transmission electron microscopy was used to visualize T4P as described (5). Briefly, 50 µl of exponentially growing *M. xanthus* cells were placed on Parafilm. A small piece of carbon-coated mica was dipped into the drop for 2 min, allowing cells to adsorb to the surface, excess liquid was soaked off, the film was placed briefly on a drop of distilled water, excess liquid was soaked off again, and the film transferred on a drop of 2% uranyl acetate (wt/vol) for 3-4 seconds and blotted dry. Transmission electron microscopy was performed on a JEOL JEM-1400 electron microscope at calibrated magnifications.

T4P shear-off assay. T4P were sheared off *M. xanthus* cells as described (6) with minor modifications. Briefly, cells were grown on 1% CTT/1.5% agar plates at 32°C for 3 days, gently harvested and resuspended in resuspension buffer (100 mM Tris-HCl pH 7.6, 100 mM NaCl). Cells were harvested for 2 min at 13,000 *g* at RT, resuspended in 200 µl SDS lysis buffer (10% (v/v) glycerol, 60 mM Tris-HCl pH 6.8, 5 mM EDTA, 2% (w/v) SDS, 100 mM DTT, 0.005% bromphenol blue) and immediately denatured at 95°C for 10 min. These samples represent whole cell lysate fractions. To shear-off T4P, cells were vortexed for 10 min and then harvested by centrifugation for 20 min at 13,000 *g* at 4°C. The supernatant was removed and kept on ice; cells were resuspended in resuspension buffer, vortexed for 5 min and harvested as described. The supernatant removed and combined with the previous supernatant. The combined supernatant was centrifuged twice for 10 min at 13,000 *g* at 4°C. Subsequently, T4P in the final supernatant were precipitated ON at 4°C by addition of PEG6000 and $MgCl_2$ to final concentrations of 2% (w/v) and 0.1 M, respectively. Whole cell fraction and sheared fraction were analyzed by immunoblot using α-PilA antibodies (7) and α-PilC antibodies (8) as a loading control.

Immunoblot analysis. Immunoblot analysis was done as described (1). Rabbit polyclonal α-PilA (7), α-PilQ, α-PilC and α-PilM (8), α-TsaP (9), α-PilO, α-PilP and α-PilN (10), α-PilB and α-PilT (5) antibodies were used together with horseradish peroxidase-conjugated goat anti-rabbit immunoglobulin G (Sigma) as secondary antibody. Mouse α-GFP antibodies (Sigma) were used together with horseradish peroxidase-conjugated sheep anti-mouse immunoglobulin G (GE Healthcare) as secondary antibody. Blots were developed using Luminata Crescendo or Forte Western HRP Substrate (Millipore) and visualized using a LAS-4000 luminescent image analyzer (Fujifilm). Proteins were separated by SDS-PAGE as described (1).

Pull-down experiments. In experiments involving MglA-His_6, the protein (final concentration: 2.1 µM) was preloaded with GTP or GDP (final concentration: 104 µM) for 30 min at RT in buffer 1 (50 mM Tris pH 7.5, 150 mM NaCl, 5% glycerol, 5 mM $MgCl_2$). To test for direct interactions between MglA-His_6 and SgmX-Strep, the two proteins were incubated in buffer 1 for 120 min at 4°C in a total volume of 500 µl (final concentrations: 2.0 µM and 1.8 µM, respectively) in the presence of the relevant nucleotide (final concentration: 100 µM). Then 100 µl of a Ni^{2+}-NTA-agarose resin previously equilibrated in buffer 1 supplemented with the relevant nucleotide were added for 120 min. The resin was washed four times with 1 ml buffer 1 containing the relevant nucleotide at 100 µM.

Proteins were eluted in 100 µl buffer 1 supplemented with 400 mM imidazole. Fractions were analyzed by SDS-PAGE as described (1).

Protein purification. All proteins were expressed in *E. coli* BL21 (DE3) (B F^- *ompT gal dcm lon* $hsdS_B(r_B^- m_B^-)$ λ(DE3 [*lacI lacUV5-T7p07 ind1 sam7 nin5*]) [$malB^+$]$_{K\text{-}12}$(λ^S)) at 18°C or 30°C. To purify His_6-tagged proteins, Ni-NTA affinity purification was used. Briefly, cells were washed in wash buffer A (50 mM Tris pH 7.5, 150 mM NaCl, 10 mM imidazole, 5% glycerol, 5mM $MgCl_2$) and resuspended in lysis buffer A (50 ml of wash buffer A supplemented with EDTA-free protease inhibitors (Complete Protease Inhibitor Cocktail Tablet EDTA-free (Roche)). Cells were lysed by French press and cell debris removed by centrifugation (48,000 *g*, 4°C, 30 min). The cleared cell lysate was pre-mixed for 1 hr with 1ml Ni^{2+}-NTA-agarose preloaded with $NiSO_4$ as described by the manufacturer and pre-equilibrated in wash buffer A and sequentially loaded on a gravity column. The column was washed with 50 column volumes of column wash buffer (50 mM Tris pH 7.5, 150 mM NaCl, 10 mM imidazole, 5mM $MgCl_2$). Proteins were eluted with elution buffer A (50 mM Tris pH 7.5, 150 mM NaCl, 5 mM $MgCl_2$, 500 mM imidazole) using a linear imidazole gradient from 50-500 mM. Fractions containing purified His_6-tagged proteins were combined and loaded onto a HiLoad 16/600 Superdex 200 pg (GE Healthcare) gel filtration column that was equilibrated with buffer 1 supplemented with 5% glycerol. Fractions containing His_6-tagged proteins were pooled, frozen in liquid nitrogen and stored at -80°C. To purify SgmX-Strep, biotin affinity purification was used. Briefly, cells were washed in wash buffer B (100 mM Tris pH 7.5, 150 mM NaCl, 5mM $MgCl_2$) and resuspended in lysis buffer B (30 ml of wash buffer D supplemented with protease inhibitors (Complete Protease Inhibitor Cocktail Tablet (Roche))). Cells were lysed was pre-mixed for 1h with 1ml Strep-Tactin®XT Superflow® (IBA-lifesciences), preequilibrated with wash buffer D and sequentially loaded on a gravity column. The column was washed with 50 column volumes of wash buffer D. Protein was eluted with elution buffer D (150 mM Tris pH 7.5, 150 mM NaCl, 2.5 mM Desthiobiotin). Elution fractions containing SgmX-Strep were loaded onto a HiLoad 16/600 Superdex 200 pg (GE Healthcare) gel filtration column that was equilibrated with buffer D supplemented with 5% flycerol. Fractions with SgmX-Strep were pooled, frozen in liquid nitrogen and stored at -80°C.

Bioinformatics. BlastP (11) and Pfam v31.0 (pfam.xfam.org) (12) were used to identify protein domains. TMHMM v2.0 (13) and SignalP v.4.1 (14) were used to test for transmembrane domains and signal sequences with default gathering thresholds, respectively. % identity/similarity between homologous proteins were calculated using EMBOSS Needle software (15) (pairwise sequence alignment). The KEGG SSDB (Sequence Similarity DataBase) database (16) was used to identify protein homologs.

Construction of plasmids: pLC51 (plasmid for generation of in-frame deletion of *sgmX*): up- (5766 A/5766 B) and downstream fragments (5766 C/5766 D) were amplified from genomic DNA of *M. xanthus* DK1622. Subsequently, the AB and CD fragments were used as template for overlapping PCR (5766 A/5766 D) to generate the AD fragment. AD fragment was digested with HindIII+EcoRI, cloned in pBJ114 and sequenced.

pIB123 (plasmid for generation of in-frame deletion of *pilT*): up- (oPilT-A/oPilT-B) and downstream fragments (oPilT-C/oPilT-D) were amplified from genomic DNA of M. xanthus DK1622/ Subsequently, the AB and CD fragments were used as template for overlapping PCR (oPilT-A/oPilT-D) to generate the AD fragment. AD fragment was digested with HindIII+EcoRI, cloned in pBJ114 and sequenced.

pAP12 (plasmid for expression of P_{nat} *pilB-mCherry* from the *attB* site): Pnat *pilB* ($PilB_{nat}$ fw / PilB rev) fragment was amplified from genomic DNA of *M. xanthus* DK1622 and digested with NdeI +BamHI. Subsequently, fragment was cloned in pNG062 carrying C-terminal *mCherry* and sequenced.

pAP16 (plasmid for expression of P_{nat} *mCherry-pilM* from the *attB* site): Pnat ($PilM_{nat}$ fw / $PilM_{nat}$ rev) and *pilM* (PilM fw/PilM rev) fragments were amplified from genomic DNA of *M. xanthus* DK1622 and digested with NdeI+BglII and XbaI+HindIII, respectively. Subsequently, fragments were cloned in pNG063 carrying N-terminal *mCherry* and sequenced.

pAP19 (plasmid for generation of in-frame deletion of *frzE*): up- (frzE A/frzE B) and downstream fragments (frzE C/ frzE D) were amplified from genomic DNA of *M. xanthus* DK1622. Subsequently, the AB and CD fragments were used as template for overlapping PCR (frzE A/ frzE D) to generate the AD fragment. AD fragment was digested with HindIII+EcoRI, cloned in pBJ114 and sequenced.

pAP34 (plasmid for expression of P_{nat} *sgmX* from the *attB* site): Pnat sgmX fragment (P_{nat}sgmX fw/ P_{nat}sgmX rev) was amplified from genomic DNA of *M. xanthus*, digested with HindIII+EcoRI, cloned in pSWU30 and sequenced.

pAP35 (plasmid for *sgmX* replacement by *sgmX-mVenus* at native site): up- (SgmX-mvA/SgmX-mvB) and downstream fragments (SgmX-mvC/SgmX-mvD) were amplified from genomic DNA of *M. xanthus* DK1622. A fragment containing *mVenus* was amplified from plasmid pLC20 carrying *mglA-mVenus* (Venus/Cherry Fw / Venus/Cherry Rv). Subsequently, AB and *mVenus* fragments were used as template for overlapping PCR (SgmX-mvA/Venus/Cherry Rv) to generate the AB-mVenus fragment. AB-mVenus and CD fragments were digested with HindIII+XbaI and XbaI+EcoRI, respectively. Fragments were cloned in pBJ114 and sequenced.

pAP37 (plasmid for *pilQ* replacement by *pilQ-sfgfp* at native site): up- (PilQ-sfGFP A/ PilQ-sfGFP B) and downstream fragments (PilQ-sfGFP C/ PilQ-sfGFP D) were amplified from genomic DNA of *M. xanthus* DK1622. A fragment containing *sfgfp* was amplified from plasmid pSC101 (10) (sfGFP Fw / sfGFP Rev). Subsequently, AB and *sfgfp* fragments were used as template for overlapping PCR (PilQ-sfGFP A/sfGFP Rev) to generate the AB-sfGFP fragment. AB-sfGFP and CD fragments were digested with HindIII+XbaI and XbaI+EcoRI, respectively. Fragments were cloned in pBJ114 and sequenced.

pAP39 (plasmid for overexpression of SgmX-Strep): *sgmX strep* fragment was amplified (SgmX-Strep fw/SgmX-Strep rev) from genomic DNA of *M. xanthus* DK1622; Strep tag

was introduced using SgmX-Strep rev primes. Fragment was digested with XbaI+HindIII, cloned in pMAT135 (derivative of pET45-b+ lacking protein tag) and sequenced.

pAP87 (plasmid for expression of P_{nat} *mCherry-pilT* from the *attB* site): Pnat ($PilT_{nat}$ fw / $PilT_{nat}$ rev) and *pilT* (PilT fw/PilT rev) fragments were amplified from genomic DNA of *M. xanthus* DK1622 and digested with NdeI+BglII and XbaI+HindIII, respectively. Subsequently, fragments were cloned in pNG063 carrying N-terminal *mCherry* and sequenced.

pAP88 (plasmid for *mglB* replacement by *mglB*$^{A64R\ G68R}$*-mCherry* at native site): up- (MglB$^{A/GR}$-mChA / MglB$^{A/GR}$-mChB) and downstream fragments (MglB$^{A/GR}$-mChC/ MglB$^{A/GR}$-mChD) were amplified from genomic DNA of *M. xanthus* SA3954. A fragment containing *mCherry* was amplified from plasmid pDK145 carrying *mglB-mCherry* (Venus/Cherry Fw / Venus/Cherry Rv). Subsequently, AB and *mCherry* fragments were used as template for overlapping PCR (MglB$^{A/GR}$-mChA/Venus/Cherry Rv) to generate the AB-mCherry fragment. AB-mCherry and CD fragments were digested with HindIII+XbaI and XbaI+EcoRI, respectively. Fragments were cloned in pBJ114 and sequenced.

pDK56 (C-terminal fusion of *mglA* with T25 fragment of *B. pertussis* adenylate cyclase) and pDK76 (N-terminal fusion of *mglA* with T18 fragment of *B. pertussis* adenylate cyclase): *mglA* (BTHMglAfw/BTHMglArvstop for pDK56 and BTHMglAfw/BTHMglArv for pDK76) was amplified from genomic DNA of *M. xanthus* DK1622. Both fragments were digested with XbaI+EcoRI, cloned into pKT25 and pUT18 vectors and sequenced.

pDK55 (C-terminal fusion of *mglB* with T25 fragment of *B. pertussis* adenylate cyclase) and pDK77 (N-terminal fusion of *mglB* with T18 fragment of *B. pertussis* adenylate cyclase): *mglB* (BTHMglBfw/BTHMglBrvstop for pDK55 and BTHMglBfw/BTHMglBrv for pDK77) was amplified from genomic DNA of *M. xanthus* DK1622. Both fragments were digested with XbaI+EcoRI, cloned into pKT25 and pUT18 vectors and sequenced.

pSC57, pSC59 (C- and N-terminal fusions of *pilB* with T25 fragment of *B. pertussis* adenylate cyclase, respectively) and pSC69, pSC70 (C- and N-terminal fusions of *pilB* with T18 fragment of *B. pertussis* adenylate cyclase, respectively): *pilB* (opilB-pKT25fw/opilB-pKT25rv for pSC57/pSC70 and opilB-pKT25fw/opilB-pKNT25rv for pSC59/pSC69) was amplified from genomic DNA of *M. xanthus* DK1622. Both fragments were digested with BamHI+EcoRI, cloned into pKT25, pKNT25, pUT18 and pUT18C vectors in proper combinations and sequenced.

pSC58, pSC60 (C- and N-terminal fusions of *pilT* with T25 fragment of *B. pertussis* adenylate cyclase, respectively) and pSC71, pSC72 (C- and N-terminal fusions of *pilT* with T18 fragment of *B. pertussis* adenylate cyclase, respectively): *pilT* (opilT-pKT25fw/opilT-pKT25rv for pSC58/pSC72 and opilT-pKT25fw/opilT-pKNT25rv for pSC60/pSC71) was amplified from genomic DNA of *M. xanthus* DK1622. Both fragments were digested with BamHI+EcoRI, cloned into pKT25, pKNT25, pUT18 and pUT18C vectors in proper combinations and sequenced.

pSC73, pSC74 (C- and N-terminal fusions of *pilM* with T25 fragment of *B. pertussis* adenylate cyclase, respectively) and pSC61, pSC62 (C- and N-terminal fusions of *pilM*

with T18 fragment of *B. pertussis* adenylate cyclase, respectively): *pilM* (opilM-pUT18fw/opilM-pUT18Crv for pSC73/pSC62 and opilM-pUT18fw/opilM-pUT18Crv for pSC74/pSC62) was amplified from genomic DNA of *M. xanthus* DK1622. Both fragments were digested with BamHI+EcoRI, cloned into pKT25, pKNT25, pUT18 and pUT18C vectors in proper combinations and sequenced.

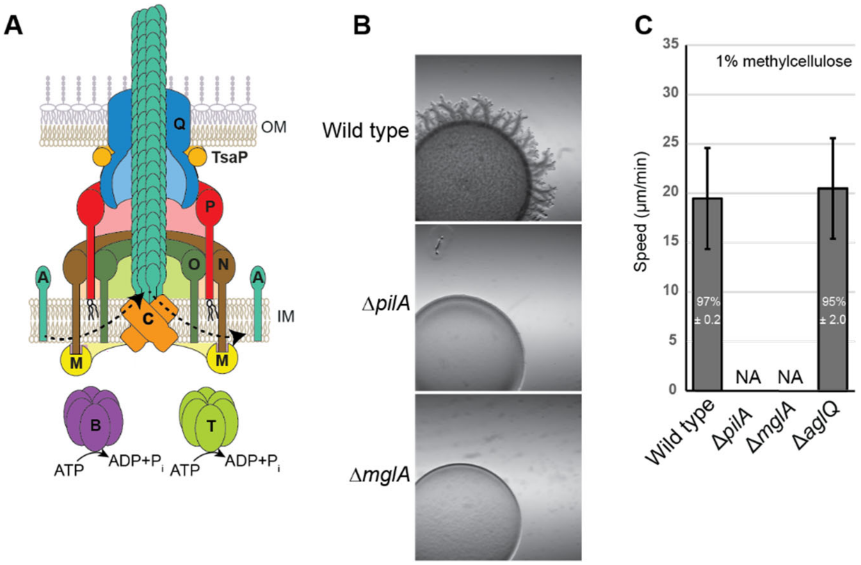

Figure S1. MglA is essential for T4P-dependent motility.
(A) Schematic of T4PM with the 10 core proteins. Proteins labelled with a single letter have the Pil prefix. Bent arrows indicate incorporation at and removal from the pilus base of PilA during extension and retraction, respectively. OM and IM, outer and inner membrane, respectively.
(B) MglA is essential for T4P-dependent motility. Strains were incubated at 32°C for 24h on 0.5% agar, 0.5% CTT. Scale bar, 1.0 mm.
(C) Speed of single cells moving by T4P-dependent motility. Histogram shows mean values, error bars indicate standard deviation (s.d.). Fraction of motile cells is indicated in white ± s.d. in the individual columns. For the WT, Δ*pilA* and Δ*aglQ* strains the cells analyzed are the same as in Fig. 5B. N = 20 cells for each strain from three independent experiments. NA, not applicable.

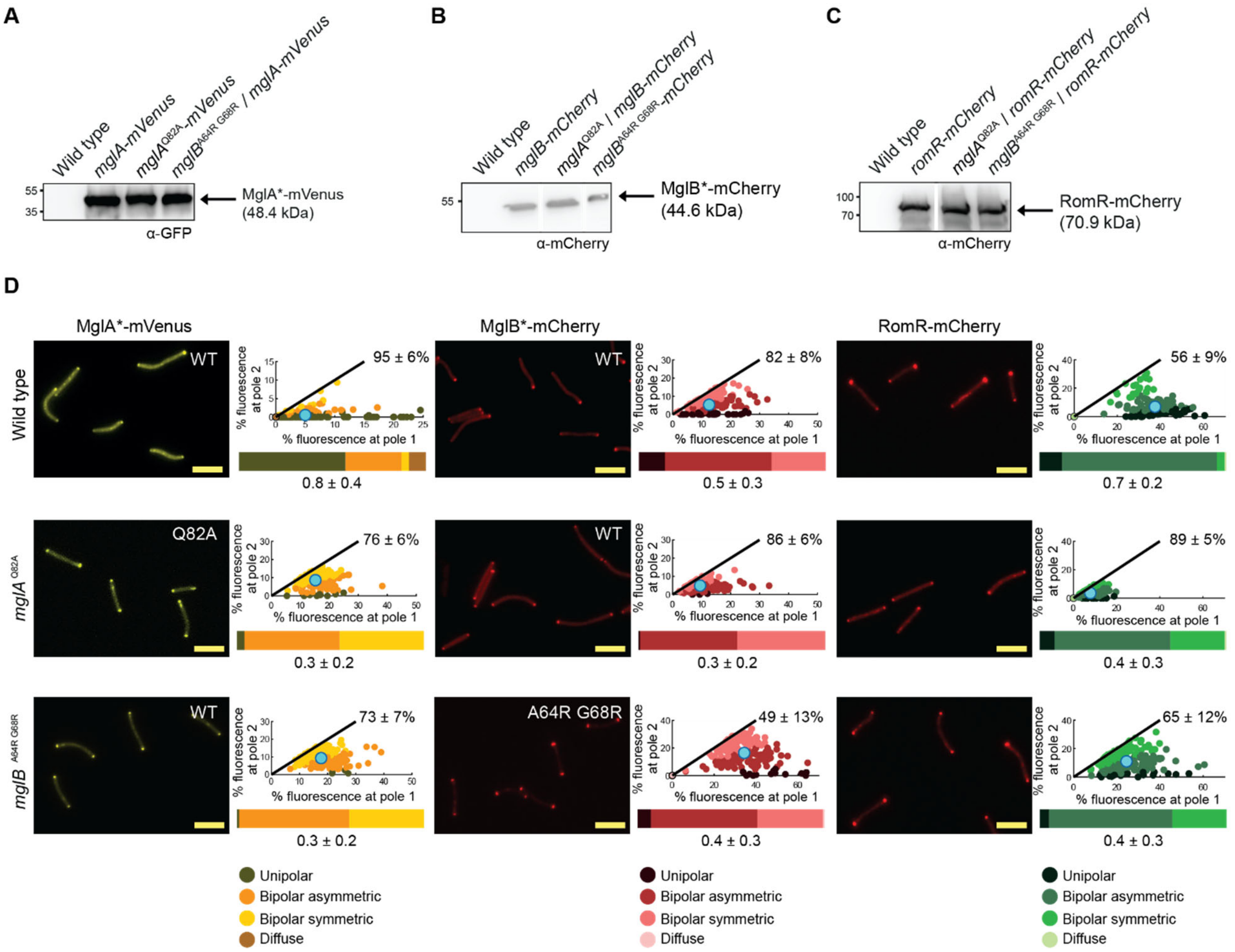

Figure S2. Accumulation and localization of MglA-mVenus variants, MglB-mCherry variants and RomR-mCherry.
(A-C) Immunoblot analysis of protein accumulation. Total cell lysates were loaded from the same number of cells per lane. Analyzed proteins are indicated on the right and molecular size markers on the left. Calculated molecular mass of monomers of the analyzed proteins is indicated on the right. In C, all samples were analyzed on the same blot and lanes were removed for presentation purposes.
(D) Localization of MglA-mVenus variants, MglB-mCherry variants and RomR-mCherry. Cells were treated, imaged and analyzed as in Fig. 3B. N= 150 cells per strain. Scale bars, 5 µm. For MglA-mVenus in WT, the data are the same as in Fig. 6C. For MglB-mCherry and RomR-mCherry in WT, the data are the same as in Fig. S10C.

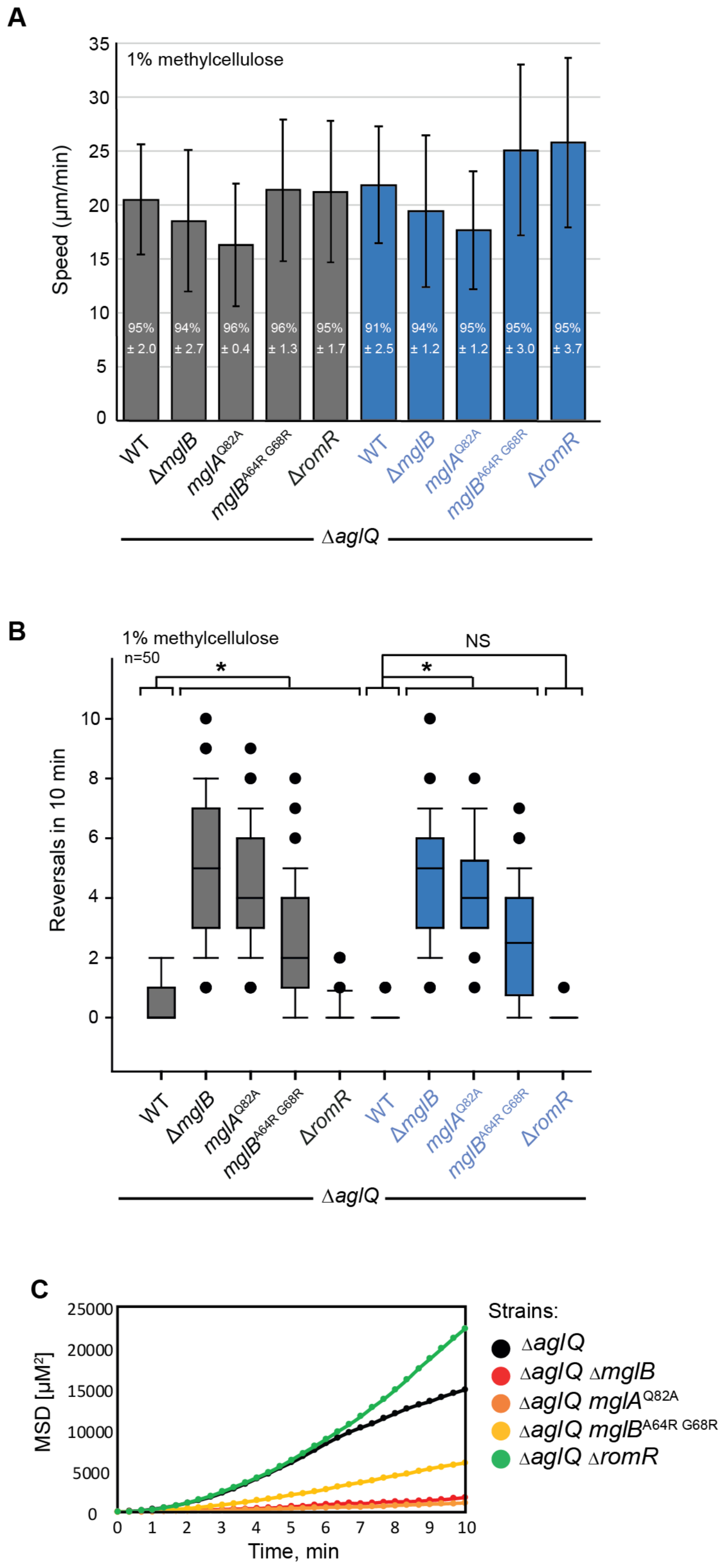

Figure S3. T4P are active at both poles in bipolarly piliated cells

(A, B) Speed and reversals of single cells moving by T4P-dependent motility. Strains

indicated in blue lack FrzE. In A, histogram shows mean values, error bars indicate s.d. and fraction of motile cells is indicated in white ± s.d. N = 20 cells for each strain from three independent experiments. For calculating the speed, only time intervals in which cells displayed movement were included. In the boxplots in B, boxes enclose the 25th and 75th percentile with the black line representing the mean, whiskers indicate the 10th and 90th percentile, and circles outliers. N=50 cells for each strain. * $P \leq 0.01$, two-sided Student's t-test. NS, not significant.

(C) Quantification of cell displacement by MSD analysis. Cells were followed for 10 min with frames captured every 20 s; N=50 cells for each strain.

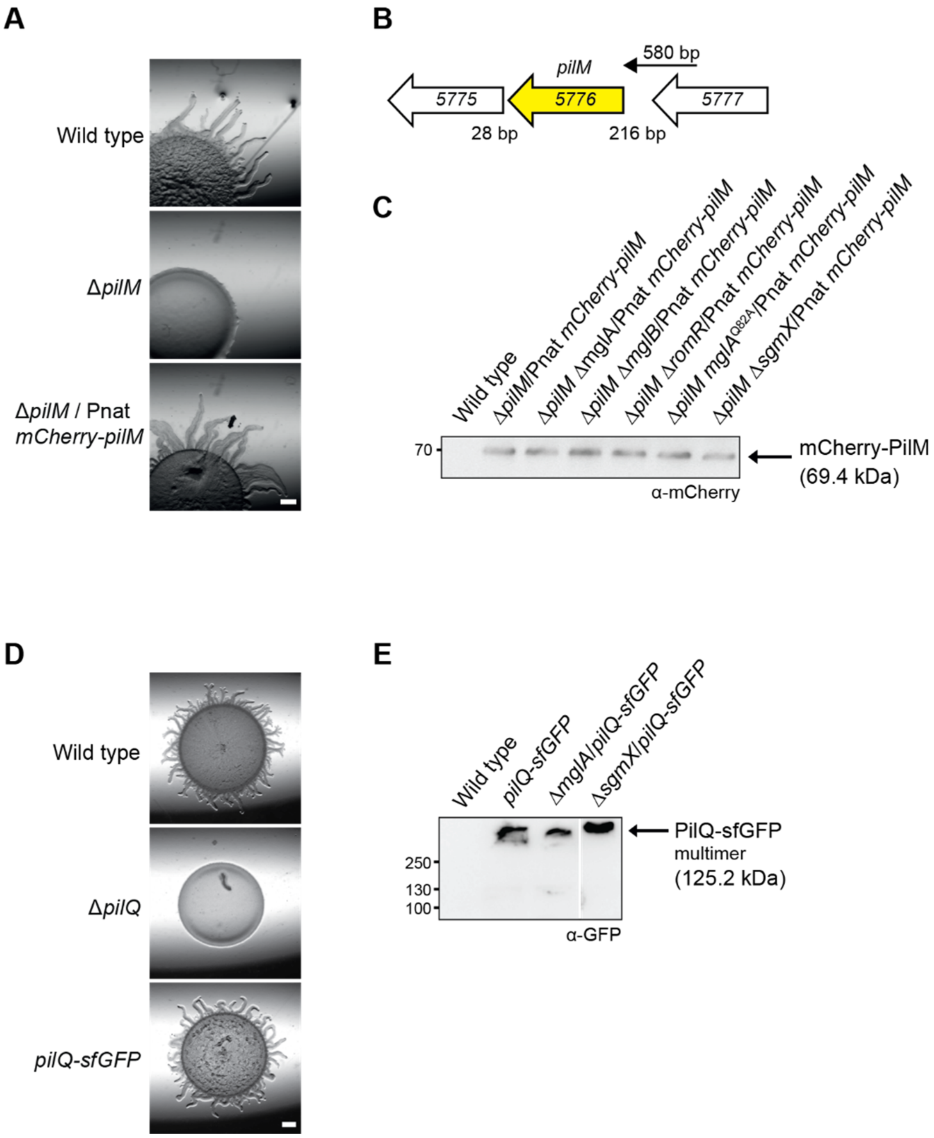

Figure S4. Analysis of mCherry-PilM and PilQ-sfGFP functionality and accumulation.

(A) P_{nat} mCherry-PilM complements T4P-dependent motility defect of Δ*pilM* mutant. Strains were incubated at 32°C for 24h on 0.5% agar, 0.5% CTT. Scale bar, 0.5 mm. *mCherry-pilM* was expressed from the native promoter in a single copy from the *attB* site.

(B) Genetic map of the *pilM* locus. Numbers below indicate distance in bp between start and stop codons of flanking genes. The arrow above indicated the native *pilM* promoter used for ectopic expression of *mCherry-pilM*. MXAN locus tags are indicated.

(C) Immunoblot analysis of mCherry-PilM accumulation. Total cell lysates were loaded from the same number of cells per lane. Calculated molecular mass of monomeric mCherry-PilM is indicated on the right and a molecular size marker on the left.

(D) PilQ-sfGFP supports T4P-dependent motility. *pilQ-sfGFP* was expressed from the native site. Strains were treated as in (A). Scale bar, 0.5 mm.

(E) Immunoblot analysis of PilQ-sfGFP accumulation. Total cell lysates were loaded from the same number of cells per lane. Molecular size markers are indicated on the left. Calculated molecular mass of monomeric PilQ-sfGFP is indicated on the right and a molecular size marker on the left. All samples were analyzed on the same blot but lanes were removed for presentation purposes.

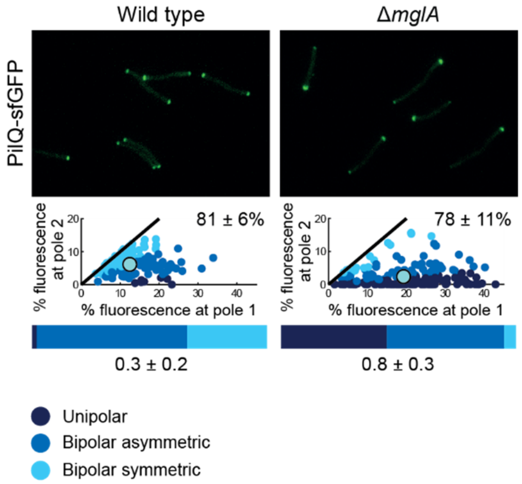

Figure S5. Localization of PilQ-sfGFP by epi-fluorescence microscopy.
Cells were treated, imaged and analyzed as in Fig. 3B.
The data for WT are the same as in Fig. 7. N = 150 cells for each strain. Scale bars, 5 μm.

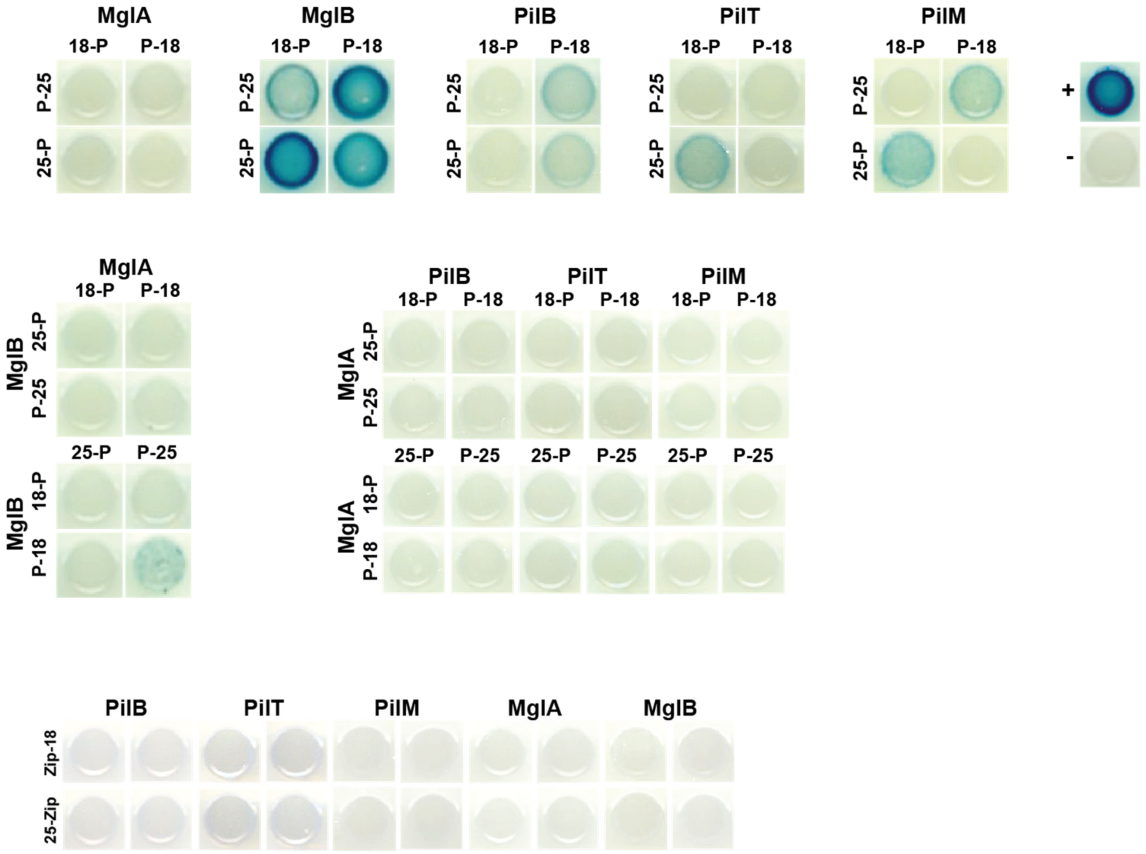

Figure S6. MglA does not detectably interact with PilB,- T or -M in a bacterial two hybrid assay.
Full-length MglA, MglB, PilB, PilT and PilM were fused to variants of the *Bordetella pertussis* adenylate cyclase and co-expressed as indicated in *E. coli* BTH101 (17). In constructs labelled 18-P and P-18 the fusions were created in the plasmids pUT18C and pUT18, respectively. In constructs labelled 25-P and P-25 the fusions were created in the plasmids pKT25 and pKNT25, respectively. In the positive control (+, upper right panel), pKT25-zip and pUT18C-zip were co-transformed. In the negative control (-, upper right panel), empty vectors encoding T25 and T18 fragments were co-transformed. In the lower panel, each MglA, MglB, PilB, PilT and PilM construct was co-expressed with pKT25-zip or pUT18C-zip to rule out possible false positive interactions. An interaction is indicated by a blue color and no interaction by a white color.

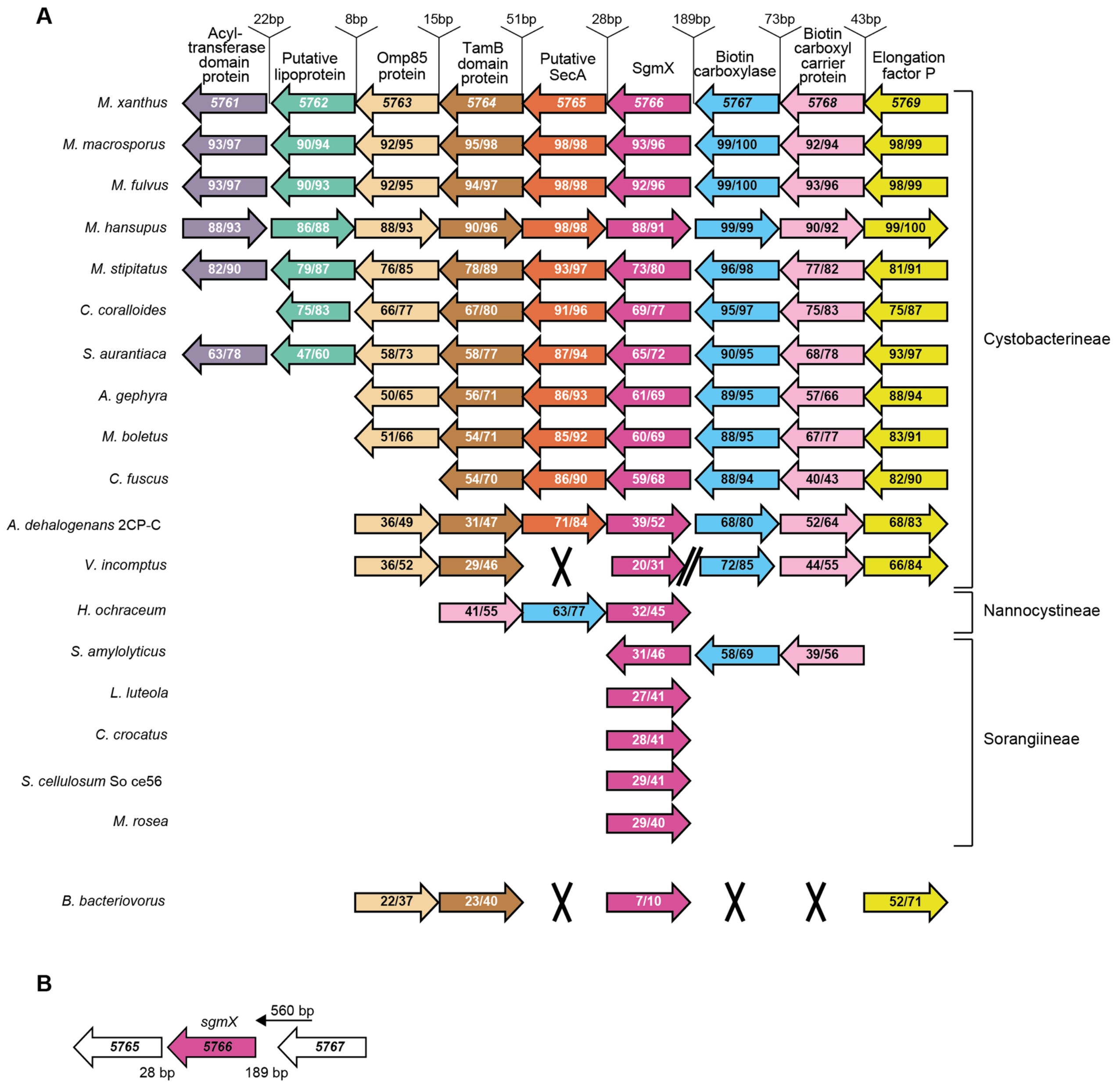

Figure S7. Analysis of the *sgmX* locus.
(A) The *sgmX* locus is largely conserved in Cystobacterineae. Distance between start and stop codons of genes in the *sgmX* locus in *M. xanthus* are indicated above. Homologous genes are indicated in the same color. %identity/%similarity are indicated for individual proteins. The *Bd2492* locus of *Bdellovibrio bacteriovorus* is included for comparison (18). Functional annotation of flanking genes is included. Together MXAN_5764 and MXAN_5763 are predicted to make up a TamA/TamB system for efficient secretion of autotransporters (18, 19). Orientation of arrows indicates direction of transcription. Cross indicates that the corresponding gene is lacking. Two parallel, slanted lines indicate the presence of two unrelated genes in *Vulgatibacter incomptus*.

(B) Genetic map of the *sgmX* locus. Numbers below indicate distance in bp between start and stop codons of flanking genes. The arrow above indicated the native *sgmX* promoter used for ectopic expression of *sgmX*. MXAN locus tags are indicated.

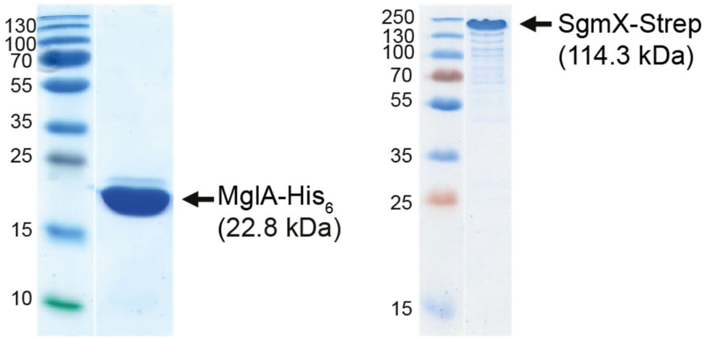

Figure S8. SDS-PAGE analysis of purified MglA-His$_6$ and SgmX-Strep.
15 ng of the indicated proteins were separated by SDS-PAGE and gels stained with Coomassie Brilliant Blue. Calculated molecular mass of the monomer of each protein is indicated on the right. Molecular markers sizes are indicated on the left. Note that SgmX-Strep separates as a protein with a size of ~233 kDa by SDS-PAGE suggesting that it is a heat- and detergent-stable dimer.

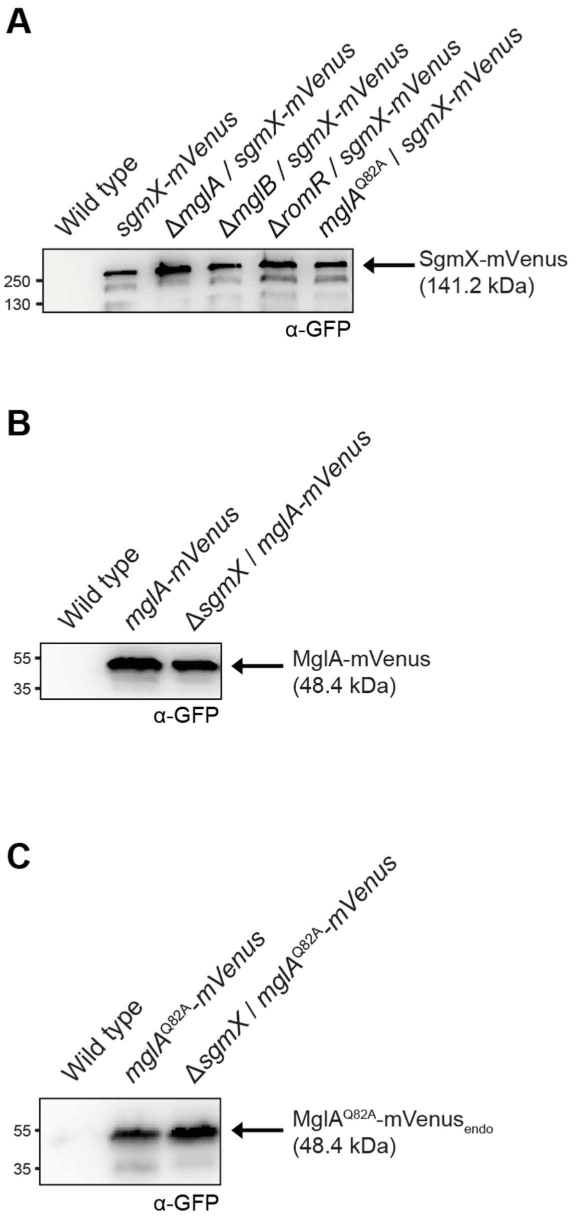

Figure S9. Accumulation of SgmX-mVenus and MglA-mVenus variants.
(A-C) Immunoblot analysis of protein accumulation. Total cell lysates were loaded from the same number of cells per lane. Analyzed proteins are indicated on the right together with the calculated molecular mass of the monomeric proteins. Molecular size markers are indicated on the left. Note that SgmX-mVenus separates as a protein with a size of ~282 kDa suggesting that it forms a heat- and detergent-stable dimer (see also Fig. S8).

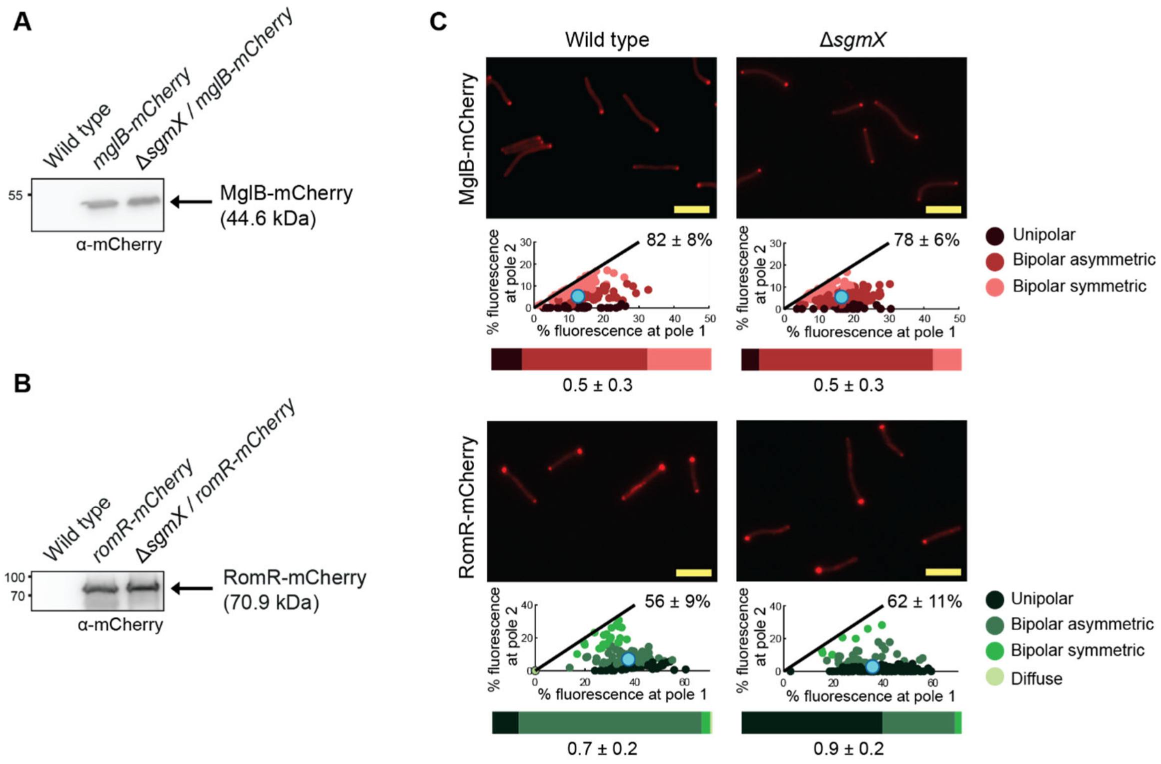

Figure S10. Accumulation and localization of MglB-mCherry and RomR-mCherry. (A, B) Immunoblot analysis of protein accumulation. Total cell lysates were loaded from the same number of cells per lane. Analyzed proteins are indicated on the right together with the calculated molecular mass of the monomeric proteins.

(C) Localization of MglB-mCherry and RomR-mCherry in the absence of SgmX by epi-fluorescence microscopy. Cells were treated, imaged and analyzed as in Fig. 3B. The data for the WT strains are the same as in Fig. S2D. Scale bars, 5 µm.

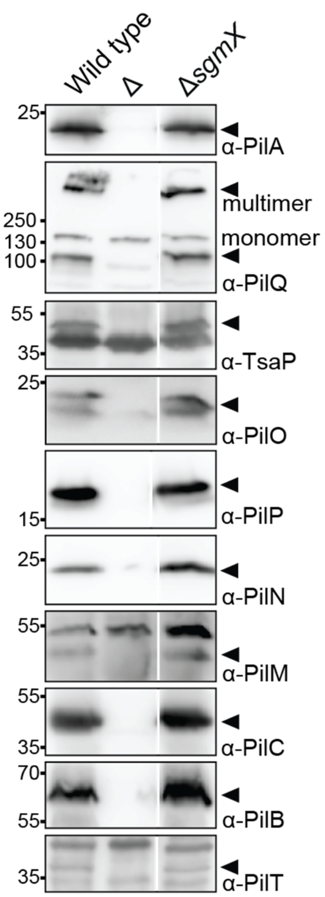

Figure S11. Accumulation of 10 core T4PM proteins in the absence of SgmX
Immunoblot analysis of accumulation of core T4PM proteins. Samples were prepared and analyzed as in Fig. 3A. For each protein, samples are from the same blot and lanes were removed for presentation purposes.

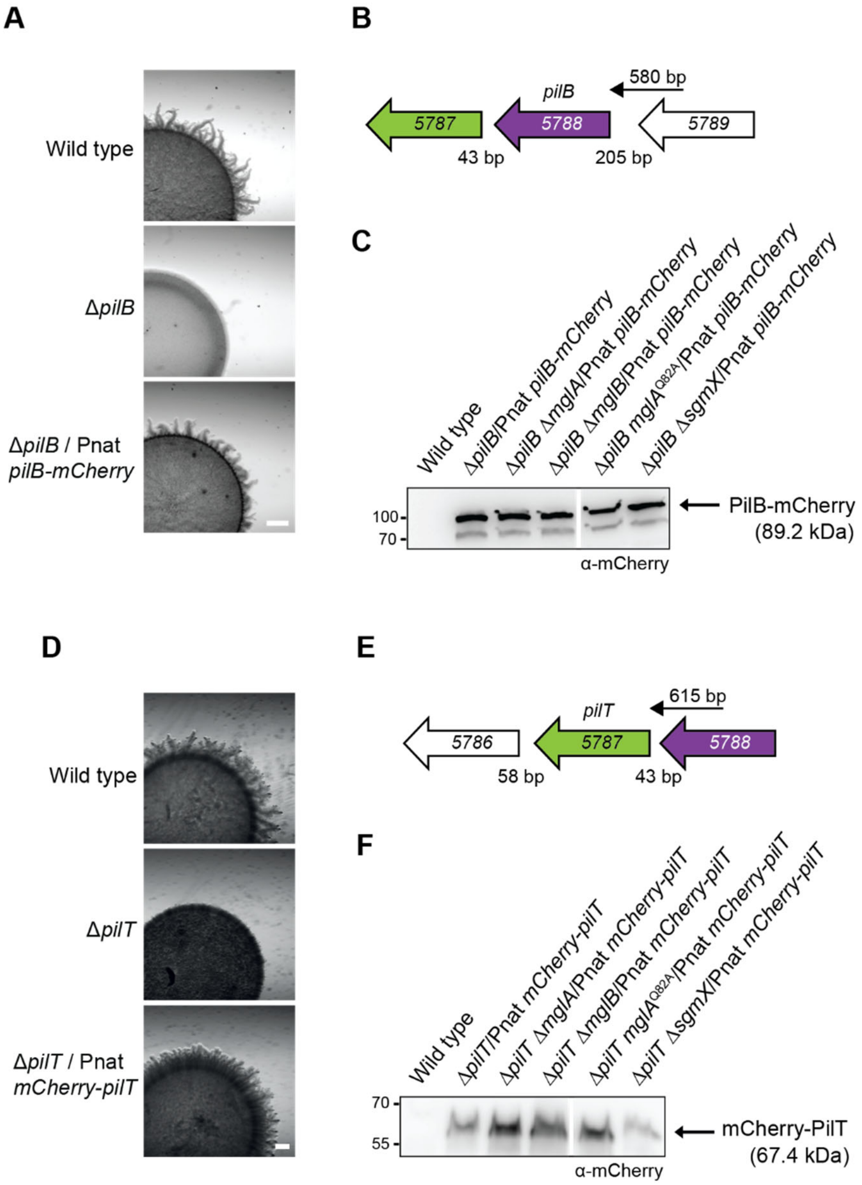

Figure S12. Analysis of PilB-mCherry and mCherry-PilT functionality and accumulation.

(A) PilB-mCherry complements T4P-dependent motility defect of a Δ*pilB* mutant. Strains were incubated at 32°C for 24h on 0.5% agar, 0.5% CTT. Scale bars, 0.5 mm. *pilB-mCherry* was ectopically expressed from the native promoter in a single copy from the *attB* site.

(B) Genetic map of the *pilB* locus. Numbers below indicate distance in bp between start and stop codons of flanking genes. The arrow above indicated the native *pilB* promoter used for ectopic expression of *pilB-mCherry*. MXAN locus tags are indicated.

(C) Immunoblot analysis of PilB-mCherry accumulation. Total cell lysates were loaded from the same number of cells per lane. Calculated molecular mass of monomeric PilB-mCherry is indicated on the right and a molecular size markers are on the left. All cell

lysates were analyzed on the same blot and lanes were removed for presentation purposes.
(D) mCherry-PilT complements T4P-dependent motility defect of a Δ*pilT* mutant. Strains were incubated at 32°C for 24h on 0.5% agar, 0.5% CTT. Scale bar, 0.5 mm. *mCherry-pilT* was ectopically expressed from the native promoter in a single copy from the *attB* site.
(E) Genetic map of the *pilT* locus. Numbers below indicate distance in bp between start and stop codons of flanking genes. The arrow above indicated the native *pilT* promoter used for ectopic expression of *mCherry-pilT*. MXAN locus tags are indicated.
(F) Immunoblot analysis of mCherry-PilT accumulation. Total cell lysates were loaded from the same number of cells per lane. Calculated molecular mass of monomeric mCherry-PilT is indicated on the right and a molecular size markers are on the left. All cell lysates were analyzed on the same blot and lanes were removed for presentation purposes.

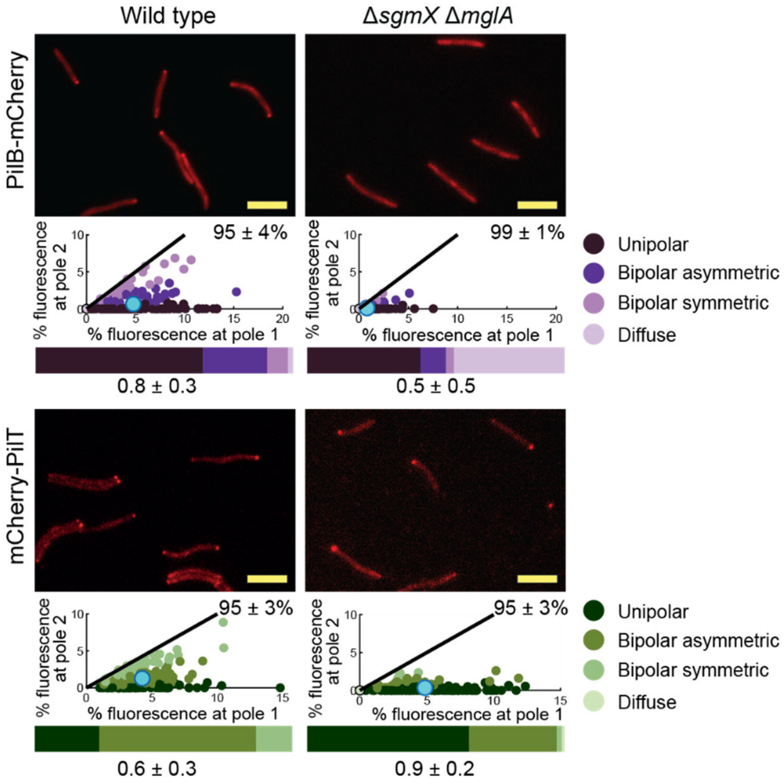

Figure S13. Localization of PilB-mCherry and mCherry-PilT.
Cells were treated, imaged and analyzed as in Fig. 3B. N = 150 cells for each strain. Scale bars, 5 μm. The data for WT are the same as in Fig. 7.

Table S1. *M. xanthus* strains used in this work

Strain	Genotype[1, 2]	Source or reference
DK1622	Wild-type	(20)
DK10410	Δ*pilA*	(21)
DK10416	Δ*pilB*	(21)
DK10417	Δ*pilC*	(21)
DK8615	Δ*pilQ*	(22)
SA3001	Δ*pilO*	(10)
SA3002	Δ*pilM*	(23)
SA3005	Δ*pilP*	(10)
SA3044	Δ*pilN*	(10)
SA6011	Δ*tsaP*	(9),
SA4420	Δ*mglA*	(24)
SA3387	Δ*mglB*	(24)
SA3300	Δ*romR*	(25)
DK10409	Δ*pilT*	(21)
SA3011	Δ*mglA* Δ*pilT*	(26)
SA3833	$mglA^{Q82A}$	(25)
SA3954	$mglB^{A64R\ G68R}$	(27)
SA5293	Δ*aglQ*	(28)
SA7101	Δ*mglB* Δ*aglQ*	This work
SA3936	Δ*mglB* Δ*romR*	(25)
SA8476	Δ*mglB* Δ*romR* Δ*aglQ*	This work
SA10743	Δ*mglB* Δ*romR* Δ*aglQ* Δ*frzE*	This work
SA7107	$mglA^{Q82A}$ Δ*aglQ*	This work
SA7124	$mglB^{A64R\ G68R}$ Δ*aglQ*	This work
SA7110	Δ*romR* Δ*aglQ*	(2)
SA7135	Δ*aglQ* Δ*frzE*	(2)
SA7136	Δ*mglB* Δ*aglQ* Δ*frzE*	This work
SA7148	$mglA^{Q82A}$ Δ*aglQ* Δ*frzE*	This work
SA8447	$mglB^{A64R\ G68R}$ Δ*aglQ* Δ*frzE*	This work
SA7147	Δ*romR* Δ*aglQ* Δ*frzE*	This work
SA7125	Δ*pilB* *attB*::P_{nat} *pilB-mCherry* (pAP12)	This work
SA7145	Δ*pilB* Δ*mglA* *attB*::P_{nat} *pilB-mCherry* (pAP12)	This work
SA8403	Δ*pilB* Δ*mglB* *attB*::P_{nat} *pilB-mCherry* (pAP12)	This work
SA7146	Δ*pilB* $mglA^{Q82A}$ *attB*::P_{nat} *pilB-mCherry* (pAP12)	This work
SA7170	Δ*pilB* Δ*sgmX* *attB*::P_{nat} *pilB-mCherry* (pAP12)	This work
SA8497	Δ*pilB* Δ*sgmX* Δ*mglA* *attB*::P_{nat} *pilB-mCherry* (pAP12)	This work
SA8465	Δ*pilT* *attB*::P_{nat} *mCherry-pilT* (pAP87)	This work
SA8466	Δ*pilT* Δ*mglA* *attB*::P_{nat} *mCherry-pilT* (pAP87)	This work
SA8467	Δ*pilT* Δ*mglB* *attB*::P_{nat} *mCherry-pilT* (pAP87)	This work
SA8468	Δ*pilT* $mglA^{Q82A}$ *attB*::P_{nat} *mCherry-pilT* (pAP87)	This work
SA8469	Δ*pilT* Δ*sgmX* *attB*::P_{nat} *mCherry-pilT* (pAP87)	This work
SA8498	Δ*pilT* Δ*sgmX* Δ*mglA* *attB*::P_{nat} *mCherry-pilT* (pAP87)	This work
SA7130	Δ*pilM* *attB*::P_{nat} *mCherry-pilM* (pAP16)	This work
SA8461	Δ*pilM* Δ*mglA* *attB*::P_{nat} *mCherry-pilM* (pAP16)	This work
SA8462	Δ*pilM* Δ*mglB* *attB*::P_{nat} *mCherry-pilM* (pAP16)	This work
SA8409	Δ*pilM* $mglA^{Q82A}$ *attB*::P_{nat} *mCherry-pilM* (pAP16)	This work
SA7174	Δ*pilM* Δ*romR* *attB*::P_{nat} *mCherry-pilM* (pAP16)	This work
SA7171	Δ*pilM* Δ*sgmX* *attB*::P_{nat} *mCherry-pilM* (pAP16)	This work
SA7192	*pilQ-sfGFP*	This work
SA7193	Δ*mglA* *pilQ-sfGFP*	This work

SA7164	Δ*sgmX*	This work
SA7175	Δ*sgmX attB*::P_{nat} *sgmX* (pAP34)	This work
SA7168	Δ*pilT* Δ*sgmX*	This work
SA8416	Δ*mglA* Δ*sgmX*	This work
SA8417	*mglA*^{Q82A} Δ*sgmX*	This work
SA8458	Δ*sgmX pilQ-sfGFP*	This work
SA7195	*sgmX-mVenus*	This work
SA7196	Δ*mglA sgmX-mVenus*	This work
SA8410	*mglA*^{Q82A} *sgmX-mVenus*	This work
SA8448	Δ*mglB sgmX-mVenus*	This work
SA8442	Δ*romR sgmX-mVenus*	This work
SA8185	*mglA-mVenus*	(2)
SA9845	Δ*sgmX mglA-mVenus*	This work
SA8455	*mglB*$^{A64R\ G68R}$ *mglA-mVenus*	This work
SA8183	*mglA*^{Q82A}-*mVenus*	(2)
SA3963	*mglB-mCherry*	(25)
SA9958	Δ*sgmX mglB-mCherry*	This work
SA10812	*mglA*^{Q82A} *mglB-mCherry*	This work
SA8471	*mglB*$^{A64R\ G68R}$-*mCherry*	This work
SA7507	*romR-mCherry*	(2)
SA9912	Δ*sgmX romR-mCherry*	This work
SA9853	*mglA*^{Q82A} *romR-mCherry*	This work
SA10014	*mglB*$^{A64R\ G68R}$ *romR-mCherry*	This work

[1] Plasmids listed in parentheses were integrated in a single copy at the Mx8 *attB* site.

[2] P_{nat} indicates that the relevant gene is expressed from the native promoter.

Table S2. Plasmids used in this work

Plasmid	Description	Reference
pSWU30	Tet^R, *attP*	(6)
pBJ114	Kan^R, *galK*	(29)
pSWU19	Kan^R, *attP*	(30)
pNG062	pSWU19 derived vector for the generation of C-terminal mCherry fusions	N. Gómez-Santos
pNG063	pSWU19 derived vector for the generation of N-terminal mCherry fusions	N. Gómez-Santos
pMAT135	pET45b+ derived vector for overexpression of proteins	A. Treuner-Lange
pTM1	Overexpression of MglA-His_6	(31)
pKT25	Vector for bacterial two hybrid system	Euromedex
pKNT25	Vector for bacterial two hybrid system	Euromedex
pUT18	Vector for bacterial two hybrid system	Euromedex
pUT18C	Vector for bacterial two hybrid system	Euromedex
pKT25-zip	pKT25 fused to leucine zipper of GCN4 yeast protein	Euromedex
pUT18C-zip	pUT18C fused to leucine zipper of GCN4 yeast protein	Euromedex
pDK56	MglA (pKT25)	This work
pDK70	MglA (pKNT25)	(32)
pDK76	MglA (pUT18)	This work
pDK75	MglA (pUT18C)	(32)
pDK55	MglB (pKT25)	This work
pDK71	MglB (pKNT25)	(32)
pDK77	MglB (pUT18)	This work
pDK74	MglB (pUT18C)	(32)
pSC57	PilB (pKT25)	This work
pSC59	PilB (pKNT25)	This work
pSC69	PilB (pUT18)	This work
pSC70	PilB (pUT18C)	This work
pSC58	PilT (pKT25)	This work
pSC60	PilT (pKNT25)	This work
pSC71	PilT (pUT18)	This work
pSC72	PilT (pUT18C)	This work
pSC73	PilM (pKT25)	This work
pSC74	PilM (pKNT25)	This work
pSC61	PilM (pUT18)	This work
pSC62	PilM (pUT18C)	This work
pLC20	pBJ114; for native site replacement of *mglA* by *mglA-mVenus*	(2)
pLC44	pBJ114; for native site replacement of *mglA* by *mglA*^{Q82A}-*mVenus*	(2)
pDK145	pBJ114; for native site replacement of *mglB* by *mglB-mCherry*	(25)
pLC32	pBJ114; for native site replacement of *romR* by *romR-mCherry*	(2)
pLC51	pBJ114; for generation of in-frame deletion of *sgmX*	This work
pBJΔ*aglQ*	pBJ114; for generation of in-frame deletion of *aglQ*	(33)
pES2	pBJ114; for generation of in-frame deletion of *mglA*	(24)
pTS08	pBJ114; for native site replacement of *mglA* by *mglA*^{Q82A}	(25)
pSL16	pBJ114; for generation of in-frame deletion of *mglA*	(27)
pSL37	pBJ114; for generation of in-frame deletion of *romR*	(25)
pIB123	pBJ114; for generation of in-frame deletion of *pilT*	This work
pAP12	pNG062; P_{nat} *pilB-mCherry*	This work

pAP16	pNG063; P_{nat} *mCherry-pilM*	This work
pAP19	pBJ114; for generation of in-frame deletion of *frzE*	This work
pAP34	pSWU30; P_{nat} *sgmX*	This work
pAP35	pBJ114; for native site replacement of *sgmX* by *sgmX-mVenus*	This work
pAP37	pBJ114; for native site replacement of *pilQ* by *pilQ-sfGFP*	This work
pAP39	pMAT135; overexpression SgmX-Strep	This work
pAP87	pNG063; P_{nat} *mCherry-pilT*	This work
pAP88	pBJ114; for native site replacement of *mglB* by $mglB^{A64R\ G68R}$*-mCherry*	This work

Table S3. Primers used in this work

Primer	Sequence	Used to construct plasmid
5766 A	ATCGAAGCTTCGAGCGTGAGTGCTCGGT	pLC51
5766 B	GCGCGCCTTAACAAGATCATCGAAGCC	
5766 C	GATCTTGTTAAGGCGCGCAAGGTCGGC	
5766 D	ATCGGAATTCGAGCGTCGACGCCGAAGC	
oPilT-A	ATCGAATTCCTCGCCCAGCGTCTGGCG	pIB123
oPilT-B	ACTCGTCCAGCGAAGCTGCGGCGGAGA	
oPilT-C	GCGAAGCTGACTCGTCCATGCAGGTCG	
oPilT-D	ATCAAGCTTCCTTGCCTTCGACACCGC	
$PilB_{nat}$ fw	ATCCATATGGATGGCACGGTGGTGACGGGC	pAP12
$PilB_{nat}$ rev	ATCAGATCTTCGTTGATGCCTCTTCCTTGA	
PilB fw	GCGCTCTAGAATGTCCGGTCGACTCGGTG	
PilB rev	ATCAAGCTTGGTTGACTAGAAGCGGTCCG	
$PilM_{nat}$ fw	ATCCATATGTCCTGAAGTCCTACGCATGG	pAP16
$PilM_{nat}$ rev	ATCAGATCTGCGTGACTCCGTCGAGAGGC	
PilM fw	ATCTCTAGAGCGAAGGGCAAACTGGTACTC	
PilM rev	ATCAAGCTTTCAGGCCAGCTTGTCGCCCG	
frzE A	ATCAAGCTTTGGCAAGGTCATCGAGACGC	pAP19
frzE B	TCTCAAGAAATCCCTCCTCGCGCAGGCCATCGACCGGC	
frzE C	TGGCCTGCGCGAGGAGGGATTTCTTGAGAGCCTCGGTG	
frzE D	ATCGAATTCGCTTCGTCCTGGGCAATGGT	
P_{nat}sgmX fw	ATCAAGCTTGAACGCCGAGGACCCGATTAC	pAP34
P_{nat}sgmX rev	ATCGAATTCCTACAGGTAGCCGACCTTGCG	
SgmX-mvA	ATCAAGCTTGCGGCGGAGGAGTGCGACGAG	pAP35
SgmX-mvB	GGAGCCGCCGCCGCCCAGGTAGCCGACCTTGCGCGC	
SgmX-mvC	ATCTCTAGATTGCACCCCGCGAGGTCCGGC	
SgmX-mvD	ATCGAATTCGAGGTCCACCTTGCTGCTCGT	
Venus/Cherry Fw	GGCGGCGGCGGCTCCATGGTGAGCAAGGGCGAG	
Venus/Cherry Rv	ATCGTCTAGATTACTTGTACAGCTCGTCCATGCC	
PilQ-sfGFP A	ATCAAGCTTAGGGTGAAGGACGTGCTGAGT	pAP37
PilQ-sfGFP B	GGAGCCGCCGCCGCCCAGAGTCTGCGCAATGGTCTG	
sfGFP Fw	GGCGGCGGCGGCTCCATGAGCAAAGGAGAAGAACTT	
sfGFP Rev	ATCTCTAGATTAGGATCCTTTGTAGAGCTC	
PilQ-sfGFP C	ATCTCTAGAGGGTTTCCCTTGCGTCCTCTT	
PilQ-sfGFP D	ATCGAATTCCTGGGTGAGGAGGTTGGCGTA	
SgmX-Strep fw	ATCTCTAGAGACAAGAACAAGATCATCGAA	pAP39
SgmX-Strep rev	ATCAAGCTTTCACTTTTCGAACTGCGGGTGGCTCCACAG GTAGCCGACCTTGCGCGC	
$PilT_{nat}$ fw	ATCCATATGGCTCCTTCCTCCGCCAGGACC	pAP87
$PilT_{nat}$ rev	ATCAGATCTGGGGGGATGTCCTTCGGGGGA	
PilT fw	ATCTCTAGAGCCAACCTGCACCAGCTCCTC	
PilT rev	ATCAAGCTTCTAACGACCACCCGCTCCCCC	
$MglB^{A/GR}$-mChA	ATCAAGCTTAGGACGCGAACGCGAAGGTGG	pAP88
$MglB^{A/GR}$-mChB	GGAGCCGCCGCCGCCCTCGCTGAAGAGGTTGTCGAT	
$MglB^{A/GR}$-mChC	ATCTCTAGACCCGGGAAGCCATGTCCTTCA	
$MglB^{A/GR}$-mChD	ATCGAATTCTCAGGTCGTAGCCCTGCTCCG	
BTHMglAfw	ATCGGTCTAGAGATGTCCTTCATCAATTACTCATCC	pDK56, pDK76
BTHMglArv	ATCGGGAATTCGAACCACCCTTCTTGAGCTCGG	
BTHMglArvstop	ATCGGGAATTCGATCAACCACCCTTCTTGAGCTCGG	

BTHMglBfw	ATCGGTCTAGAGATGGGCACGCAACTGGTGATG	pDK55, pDK77
BTHMglBrv	ATCGGGAATTCGACTCGCTGAAGAGGTTGTCGATATCG	
BTHMglBrvstop	ATCGGGAATTCGATTACTCGCTGAAGAGGTTGTCGATATCG	
opilB-pKT25fw	ATCGGGGATCCCATGTCCGGTCGACTCGGT	pSC57, pSC59, pSC69, pSC70
opilB-pKT25rv	ATCGGGAATTCCTAGAAGCGGTCCGGGGC	
opilB-pKNT25rv	ATCGGGAATTCGGGAAGCGGTCCGGGGCGGT	
opilT-pKT25fw	ATCGGGGATCCCGTGGCCAACCTGCACCAG	pSC58, pSC60, pSC71, pSC72
opilT-pKT25rv	ATCGGGAATTCCTAACGACCACCCGCTCC	
opilT-pKNT25rv	ATCGGGAATTCGGACGACCACCCGCTCCCCC	
opilM-pUT18fw	ATCGGGGATCCCATGGTGCGAGGCTCCCGT	pSC61, pSC62, pSC73, pSC74
opilM-pUT18rv	ATCGGGAATTCGGGGCCAGCTTGTCGCCCGG	
opilM-pUT18Crv	ATCGGGAATTCTCAGGCCAGCTTGTCGCC	

SI References

1. J. Sambrook, D. W. Russell, *Molecular cloning: A Laboratory Manual* (Cold Spring Harbor Laboratory Press, Cold Spring Harbor, N.Y., ed. 3rd, 2001).

2. D. Szadkowski *et al.*, Spatial control of the GTPase MglA by localized RomR/RomX GEF and MglB GAP activities enables *Myxococcus xanthus* motility. *Nat. Microbiol.* **4**, 1344-1355 (2019).

3. W. Shi, D. R. Zusman, The two motility systems of *Myxococcus xanthus* show different selective advantages on various surfaces. *Proc. Natl. Acad. Sci. USA* **90**, 3378-3382 (1993).

4. C. A. Schneider, W. S. Rasband, K. W. Eliceiri, NIH Image to ImageJ: 25 years of image analysis. *Nat. Methods* **9**, 671-675 (2012).

5. V. Jakovljevic, S. Leonardy, M. Hoppert, L. Søgaard-Andersen, PilB and PilT are ATPases acting antagonistically in type IV pili function in *Myxococcus xanthus*. *J. Bacteriol.* **190**, 2411-2421 (2008).

6. S. S. Wu, D. Kaiser, Regulation of expression of the *pilA* gene in *Myxococcus xanthus*. *J. Bacteriol.* **179**, 7748-7758 (1997).

7. Y. Li, R. Lux, A. E. Pelling, J. K. Gimzewski, W. Shi, Analysis of type IV pilus and its associated motility in *Myxococcus xanthus* using an antibody reactive with native pilin and pili. *Microbiology* **151**, 353-360 (2005).

8. I. Bulyha *et al.*, Regulation of the type IV pili molecular machine by dynamic localization of two motor proteins. *Mol. Microbiol.* **74**, 691–706 (2009).

9. K. Siewering *et al.*, Peptidoglycan-binding protein TsaP functions in surface assembly of type IV pili. *Proc. Natl. Acad. Sci. USA* **111**, E953-961 (2014).

10. C. Friedrich, I. Bulyha, L. Søgaard-Andersen, Outside-in assembly pathway of the type IV pili system in *Myxococcus xanthus*. *J Bacteriol* **196**, 378-390 (2014).

11. G. M. Boratyn *et al.*, BLAST: a more efficient report with usability improvements. *Nucleic Acids Res* **41**, W29-33 (2013).

12. R. D. Finn *et al.*, The Pfam protein families database: towards a more sustainable future. *Nucleic Acids Research* **44**, D279-D285 (2015).

13. E. L. Sonnhammer, G. von Heijne, A. Krogh, A hidden Markov model for predicting transmembrane helices in protein sequences. *Proc Int Conf Intell Syst Mol Biol* **6**, 175-182 (1998).

14. T. N. Petersen, S. Brunak, G. von Heijne, H. Nielsen, SignalP 4.0: discriminating signal peptides from transmembrane regions. *Nat. Methods* **8**, 785-786 (2011).

15. W. Li *et al.*, The EMBL-EBI bioinformatics web and programmatic tools framework. *Nucl Acids Res* **43**, W580-W584 (2015).

16. M. Kanehisa, S. Goto, KEGG: kyoto encyclopedia of genes and genomes. *Nucleic Acids Res* **28**, 27-30 (2000).

17. G. Karimova, J. Pidoux, A. Ullmann, D. Ladant, A bacterial two-hybrid system based on a reconstituted signal transduction pathway. *Proc. Natl. Acad. Sci. USA* **95**, 5752-5756 (1998).

18. D. S. Milner *et al.*, Ras GTPase-like protein MglA, a controller of bacterial social-motility in Myxobacteria, has evolved to control bacterial predation by *Bdellovibrio*. *PLOS Genetics* **10**, e1004253 (2014).

19. J. Selkrig *et al.*, Discovery of an archetypal protein transport system in bacterial outer membranes. *Nat Struct Mol Biol* **19**, 506-510 (2012).

20. D. Kaiser, Social gliding is correlated with the presence of pili in *Myxococcus xanthus*. *Proc. Natl. Acad. Sci. USA* **76**, 5952-5956 (1979).

21. S. S. Wu, J. Wu, D. Kaiser, The *Myxococcus xanthus pilT* locus is required for social gliding motility although pili are still produced. *Mol. Microbiol.* **23**, 109-121 (1997).

22. D. Wall, P. E. Kolenbrander, D. Kaiser, The *Myxococcus xanthus pilQ* (*sglA*) gene encodes a secretin homolog required for type IV pilus biogenesis, social motility, and development. *J Bacteriol* **181**, 24-33 (1999).

23. I. Bulyha *et al.*, Regulation of the type IV pili molecular machine by dynamic localization of two motor proteins. *Mol Microbiol* **74**, 691-706 (2009).

24. S. Leonardy *et al.*, Regulation of dynamic polarity switching in bacteria by a Ras-like G-protein and its cognate GAP. *EMBO J.* **29**, 2276-2289 (2010).

25. D. Keilberg, K. Wuichet, F. Drescher, L. Søgaard-Andersen, A response regulator interfaces between the Frz chemosensory system and the MglA/MglB GTPase/GAP module to regulate polarity in *Myxococcus xanthus*. *PLOS Genet.* **8**, e1002951 (2012).

26. I. Bulyha *et al.*, Two small GTPases act in concert with the bactofilin cytoskeleton to regulate dynamic bacterial cell polarity. *Dev. Cell* **25**, 119–131 (2013).

27. M. Miertzschke *et al.*, Structural analysis of the Ras-like G protein MglA and its cognate GAP MglB and implications for bacterial polarity. *EMBO J.* **30**, 4185-4197 (2011).

28. B. Jakobczak, D. Keilberg, K. Wuichet, L. Søgaard-Andersen, Contact- and protein transfer-dependent stimulation of assembly of the gliding motility machinery in *Myxococcus xanthus*. *PLOS Genet* **11**, e1005341 (2015).

29. B. Julien, A. D. Kaiser, A. Garza, Spatial control of cell differentiation in *Myxococcus xanthus*. *Proc. Natl. Acad. Sci. USA* **97**, 9098-9103 (2000).

30. S. S. Wu, D. Kaiser, Genetic and functional evidence that type IV pili are required for social gliding motility in *Myxococcus xanthus*. *Mol. Microbiol.* **18**, 547-558 (1995).

31. Y. Zhang, M. Franco, A. Ducret, T. Mignot, A bacterial Ras-like small GTP-binding protein and its cognate GAP establish a dynamic spatial polarity axis to control directed motility. *PLOS Biol* **8**, e1000430 (2010).

32. A. L. McLoon *et al.*, MglC, a paralog of *Myxococcus xanthus* GTPase-Activating Protein MglB, plays a divergent role in motility regulation. *J. Bacteriol.* **198**, 510-520 (2016).

33. M. Sun, M. Wartel, E. Cascales, J. W. Shaevitz, T. Mignot, Motor-driven intracellular transport powers bacterial gliding motility. *Proc. Natl. Acad Sci. USA* **108**, 7559-7564 (2011).
